# Loss of ASAP1 in mice impairs adipogenic and osteogenic differentiation of mesenchymal progenitor cells through dysregulation of FAK/Src and AKT signaling

**DOI:** 10.1371/journal.pgen.1008216

**Published:** 2019-06-27

**Authors:** Caroline Schreiber, Supriya Saraswati, Shannon Harkins, Annette Gruber, Natascha Cremers, Wilko Thiele, Melanie Rothley, Diana Plaumann, Claudia Korn, Olivier Armant, Hellmut G. Augustin, Jonathan P. Sleeman

**Affiliations:** 1 European Center for Angioscience (ECAS), Medical Faculty of Mannheim, University of Heidelberg, Mannheim, Germany; 2 Institute for Toxicology and Genetics, KIT Campus Nord, Karlsruhe, Germany; 3 German Cancer Research Center (DKFZ-ZMBH-Alliance), Heidelberg, Germany; Stanford University School of Medicine, UNITED STATES

## Abstract

ASAP1 is a multi-domain adaptor protein that regulates cytoskeletal dynamics, receptor recycling and intracellular vesicle trafficking. Its expression is associated with poor prognosis for a variety of cancers, and promotes cell migration, invasion and metastasis. Little is known about its physiological role. In this study, we used mice with a gene-trap inactivated ASAP1 locus to study the functional role of ASAP1 *in vivo*, and found defects in tissues derived from mesenchymal progenitor cells. Loss of ASAP1 led to growth retardation and delayed ossification typified by enlarged hypertrophic zones in growth plates and disorganized chondro-osseous junctions. Furthermore, loss of ASAP1 led to delayed adipocyte development and reduced fat depot formation. Consistently, deletion of ASAP1 resulted in accelerated chondrogenic differentiation of mesenchymal cells *in vitro*, but suppressed osteo- and adipogenic differentiation. Mechanistically, we found that FAK/Src and PI3K/AKT signaling is compromised in *Asap1*^*GT*/GT^ MEFs, leading to impaired adipogenic differentiation. Dysregulated FAK/Src and PI3K/AKT signaling is also associated with attenuated osteogenic differentiation. Together these observations suggest that ASAP1 plays a decisive role during the differentiation of mesenchymal progenitor cells.

## Author summary

Mesenchymal progenitor cells are capable of differentiating into a number of lineages including osteoblasts, chondrocytes and adipocytes, and have therefore attracted interest for their potential application in regenerative medicine. Furthermore, defects in mesenchymal progenitor cell differentiation are considered to contribute to various diseases including metabolic syndrome, obesity and osteoporosis. In this study, we analyzed mice deficient in the multi-adaptor protein ASAP1, which has been implicated in tumor progression and metastasis. These mice display growth retardation, and a delayed development of bone and fat tissue. Consistently, mesenchymal progenitor cells deficient in ASAP1 exhibited enhanced differentiation into chondrocytes, but impaired differentiation into adipocytes and osteoblasts. Together these observations suggest that ASAP1 plays a decisive role during the differentiation of mesenchymal stem cells, which may be relevant for a number of diseases such as cancer.

## Introduction

Mesenchymal stem cells (MSCs) are multipotent progenitor cells capable of differentiating into a number of lineages including osteoblasts, chondrocytes and adipocytes. Due to their multipotency, MSCs have attracted interest for their potential application in regenerative medicine. In addition, impairment of mesenchymal differentiation is considered to contribute to many diseases, including metabolic syndrome, obesity and osteoporosis [1–3].

Mesenchymal differentiation is regulated by key transcription factors that are specific for each lineage. Osteogenic differentiation is driven by the transcription factor Runx2, and leads to the formation of new bone in the process of osteogenesis. Together with the osteoblast-specific transcription factor osterix, Runx2 induces the expression of matrix proteins such as osteocalcin, osteopontin, bone sialoprotein and collagen I, which leads to the maturation of osteoblasts and subsequent mineralization of the bone matrix [4, 5].

Chondrogenesis involves the differentiation of mesenchymal progenitor cells into chondrocytes and the formation of cartilage, and is initiated by the condensation of mesenchymal cells. The transcription factor Sox9 is required for chondrogenic determination, and drives the expression of early chondrocyte-specific genes such as collagen 2a and aggrecan [6, 7]. Later, Sox9 is down-regulated and the transcription factor Runx2 promotes hypertrophy, which is marked by expression of Indian hedgehog (Ihh), collagen X and MMP13 [8].

The formation of adipocytes from mesenchymal progenitor cells is driven by the transcription factor PPARγ, the master regulator of adipogenesis [9–11]. Together with the CCAAT-enhancer-binding protein α (C/EBPα), PPARγ activates the transcription of genes expressed in mature adipocytes such as adiponectin, perilipin and Glut4 [9].

In the embryo, most bones are formed by endochondral ossification, in which chondrocytes generate a cartilage model that guides future bone formation, and form the growth plate, a highly organized zone of chondrocyte proliferation, maturation and hypertrophy [8, 12]. Mesenchymal cells in the perichondrium surrounding the cartilage differentiate into osteoprogenitor cells [13]. Together with osteoclasts, blood vessels and hematopoietic cells, they invade into the hypertrophic cartilage to form the primary ossification center, which leads to replacement of the cartilage with bone marrow and mineralized bone.

Mesenchymal differentiation is modulated by a complex network of intracellular and extracellular signals. Intracellularly, focal adhesion kinase (FAK) and the non-receptor kinase Src are pivotal signaling molecules that coordinate cytoskeletal changes during lineage differentiation, and which are functionally implicated in adipogenic, osteogenic and chondrogenic differentiation [14–19]. Thus, inhibition of FAK signaling by siRNA or chemical inhibitors prevents adipogenic differentiation [14]. Similarly, inhibition of Src-family tyrosine kinases prevents fat accumulation [15]. Furthermore, FAK promotes osteogenic differentiation while Src inhibits osteogenesis [16, 17].

FAK activation is regulated by growth factor receptors or integrins at the plasma membrane. Integrin-ECM interactions promote dimerization of FAK and its auto-phosphorylation at Y397 [20]. This facilitates binding of Src to FAK, and allows the phosphorylation of the FAK kinase domain by Src. Active FAK in turn phosphorylates Src at Y416 [21]. FAK phosphorylated at Y397 also interacts with the regulatory subunit of phosphoinositide 3-kinase (PI3K), leading to activation of the serine/threonine kinase AKT [22]. In addition, activation of the FAK/Src complex stimulates other signaling cascades, including MEK/ERK, p38 and GTPases of the Rho family, and promotes migration, proliferation and differentiation [21].

ASAP1, also called AMAP1 or DDEF1, is a multi-domain adaptor protein involved in cytoskeletal re-arrangement, cell motility and metastasis. ASAP1 is an Arf GTP activating protein with activity for ADP-ribosylation factors Arf1 and Arf5, and is a downstream effector of Arf6 [23–25]. ASAP1 contains a BAR domain, as well as a proline-rich domain and a SH3 domain that allow ASAP1 to interact with a variety of intracellular regulatory proteins including FAK, Src, cortactin, and IKKβ [23, 25–27]. Overexpression of ASAP1 promotes focal adhesion formation and enhanced migration, whereas loss of ASAP1 inhibits podosome and invadopodia formation, and reduces spreading on fibronectin [26, 28–30]. Furthermore, ASAP1 regulates integrin β1 recycling, and thereby promotes cancer cell invasion [31]. Previously, we have shown that ASAP1 promotes the migration and metastasis of pancreatic tumor cells *in vivo* and correlates with poor survival of colorectal patients [32]. Furthermore, ASAP1 expression is upregulated in a variety of other tumors including breast, prostate carcinomas and uveal melanomas [25, 33, 34].

Here, we investigated the physiological role of ASAP1 by analyzing mice with a gene-trap-inactivated ASAP1 locus, and found that ASAP1 is strongly expressed during embryonic development. Loss of ASAP1 *in vivo* resulted in partial perinatal lethality, delayed ossification, and a decreased bodyweight associated with reduced amounts of fat tissue. Using primary MEF cultures *in vitro*, we found that loss of ASAP1 suppressed osteo- and adipogenic differentiation, but promoted chondrogenic differentiation. Mechanistically, ASAP-deficient MEFs failed to activate FAK/Src and AKT signaling appropriately upon induction of adipogenesis or osteogenesis. These data identify ASAP1 as a pivotal intracellular regulator of mesenchymal progenitor cell differentiation that acts through the regulation of FAK/Src-AKT signaling.

## Results

### Generation and analysis of ASAP1 gene-trap mice

To determine the physiological role of ASAP1 *in vivo*, we used mice bearing the gene-trap targeting vector pGT0Lxf integrated into the ASAP1 locus. This vector contains a splice-acceptor sequence upstream of a β-geo reporter gene and a polyA tail. Genomic integration results in disruption of the coding sequence of the targeted gene and the concomitant expression of the β-geo marker, allowing cells in which the targeted gene is expressed to be detected using X-gal staining [35, 36].

**Fig 1:**
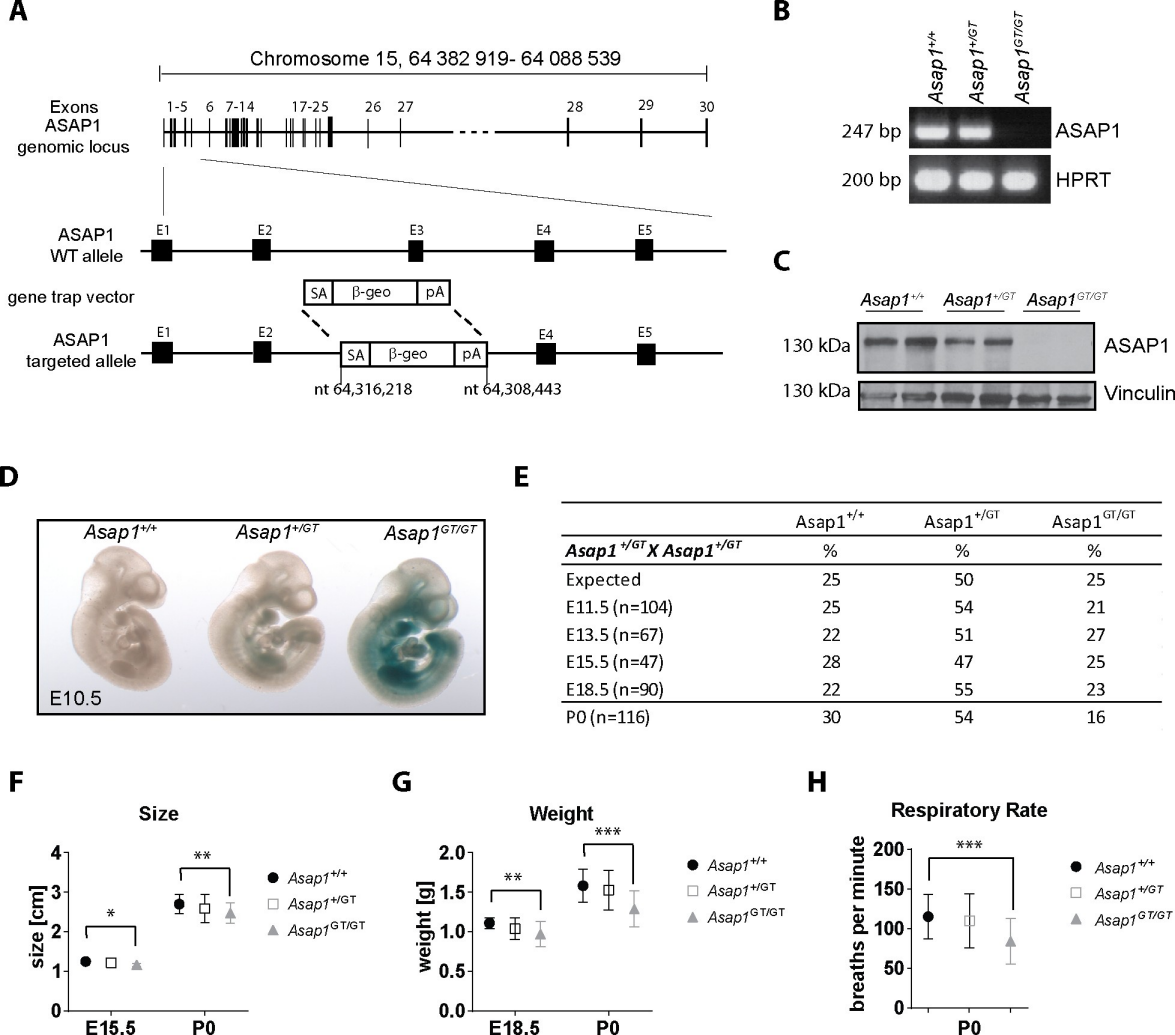
Characterization of ASAP1 gene-trap mice. **(A)** Schematic diagram showing ASAP1 gene locus (upper part) and integration of the pGT0Lxf gene-trap vector (lower part). SA: splice acceptor; β-geo: β-geo reporter cassette; pA: polyadenylation signal; E: exon; nt: nucleotide. **(B and C)** ASAP1 expression is lost in *Asap1*^*GT*/GT^ embryos. mRNA and protein expression of ASAP1 was determined in E11.5 *Asap1*^+/+^, *Asap1*^+/GT^ and *Asap1*^*GT*/GT^ embryos using semi-quantitative RT-PCR and Western blot. Two embryos per genotype were analyzed for protein expression. HPRT and vinculin served as loading controls. (**D**) X-Gal staining of E10.5 *Asap1*^+/+^, *Asap1*^+/GT^ and *Asap1*^*GT*/GT^ embryos. (**E**) Table showing differences in Mendelian ratios at birth. Heterozygous mice were intercrossed and genotype of progeny was analyzed at embryonic day E11.5, E13.5, E15.5 and E18.5 and at day of birth, P0. Numbers indicate percentage of genotypes among embryos analyzed. **(F)** Analysis of embryo size at E15.5 (*Asap1*^+/+^ n = 8, *Asap1*^+/GT^ n = 15 and *Asap1*^*GT*/GT^ n = 8) and P0 (*Asap1*^+/+^ n = 35, *Asap1*^+/GT^ n = 62 and *Asap1*^*GT*/GT^ n = 19) measured from crown to rump. (**G**) Analysis of weight at E18.5 (*Asap1*^+/+^ n = 20, *Asap1*^+/GT^ n = 51 and *Asap1*^*GT*/GT^ n = 27) and P0 (*Asap1*^+/+^ n = 35, *Asap1*^+/GT^ n = 62 and *Asap1*^*GT*/GT^ n = 19). (**H**) Analysis of respiratory rate at P0. Three independent measurements per embryo were taken (*Asap1*^+/+^ n = 35, *Asap1*^+/GT^ n = 62 and *Asap1*^*GT*/GT^ n = 19). For F-H, graphs show mean ± SD. Significance was calculated using the Student’s t-test. *, p < 0.05, ** p < 0.005, *** p < 0.001.

Whole genome sequencing was used to confirm specific targeting of the ASAP1 locus, which revealed integration of the gene-trap vector into the ASAP1 gene on chromosome 15 between nucleotides 64,308,443 and 64,316,218, and a concurrent deletion of the corresponding part of the ASAP1 gene including exon 3 (nt 64,312,349–64,312,475) (Fig 1A). The sequence information was used to develop a genomic PCR amplification strategy for genotyping (S1 Fig). No other pGT0Lxf vector integration sites were found in the genome sequence, verifying unique disruption of the ASAP1 locus in the mice.

Heterozygous *Asap1*^*GT*^ mice were intercrossed to generate E10.5 and E11.5 embryos that were genotyped and subsequently analyzed by RT-PCR, Western blot and X-Gal staining. The insertion of the gene-trap vector resulted in the ablation of ASAP1 transcripts and protein expression (Fig 1B and 1C), while expression of *lacZ* could be detected in *Asap1*^+/GT^ and *Asap1*^*GT*/GT^ mice using X-Gal staining (Fig 1D). Using primer pairs covering different parts of the ASAP1 mRNA, we verified that the 31 exons of the ASAP1 locus 3’ to the insertion site of the gene-trap vector were not expressed (S1 Fig).

### *Asap1*^GT/GT^ mice are susceptible to perinatal lethality, and display growth retardation

Intercrosses of heterozygous *Asap1*^+/GT^ mice were used to produce *Asap1*^+/+^, *Asap1*^+/GT^ and *Asap1*^*GT*/GT^ mice. Analysis of the Mendelian ratio of 3-week-old litters showed that *Asap1*^*GT*/GT^ mice were significantly underrepresented in the progeny (S1 Fig). Analysis of embryos from E11.5 up to birth revealed no differences in the Mendelian ratio between *Asap1*^+/+^, *Asap1*^+/GT^ and *Asap1*^*GT*/GT^ embryos during gestation. At P0, however, the number of *Asap1*^*GT*/GT^ animals was substantially reduced, suggesting that *Asap1*^*GT*/GT^ neonates died during or directly after birth (Fig 1E).

From E15.5 onwards, *Asap1*^*GT*/GT^ embryos exhibited a reduced size compared to *Asap1*^+/+^ embryos (Fig 1F). At E18.5 and P0, *Asap1*^*GT*/GT^ embryos also had a significantly lower body weight compared to *Asap1*^+/+^ embryos (Fig 1G). Furthermore, *Asap1*^*GT*/GT^ neonates displayed a reduced respiratory rate during the first day after birth (Fig 1H). The respiratory rate recovered in *Asap1*^*GT*/GT^ neonates that survived the first day after birth, and the surviving pups reached adulthood.

### ASAP1 is expressed in cartilage tissue and the heart

**Fig 2:**
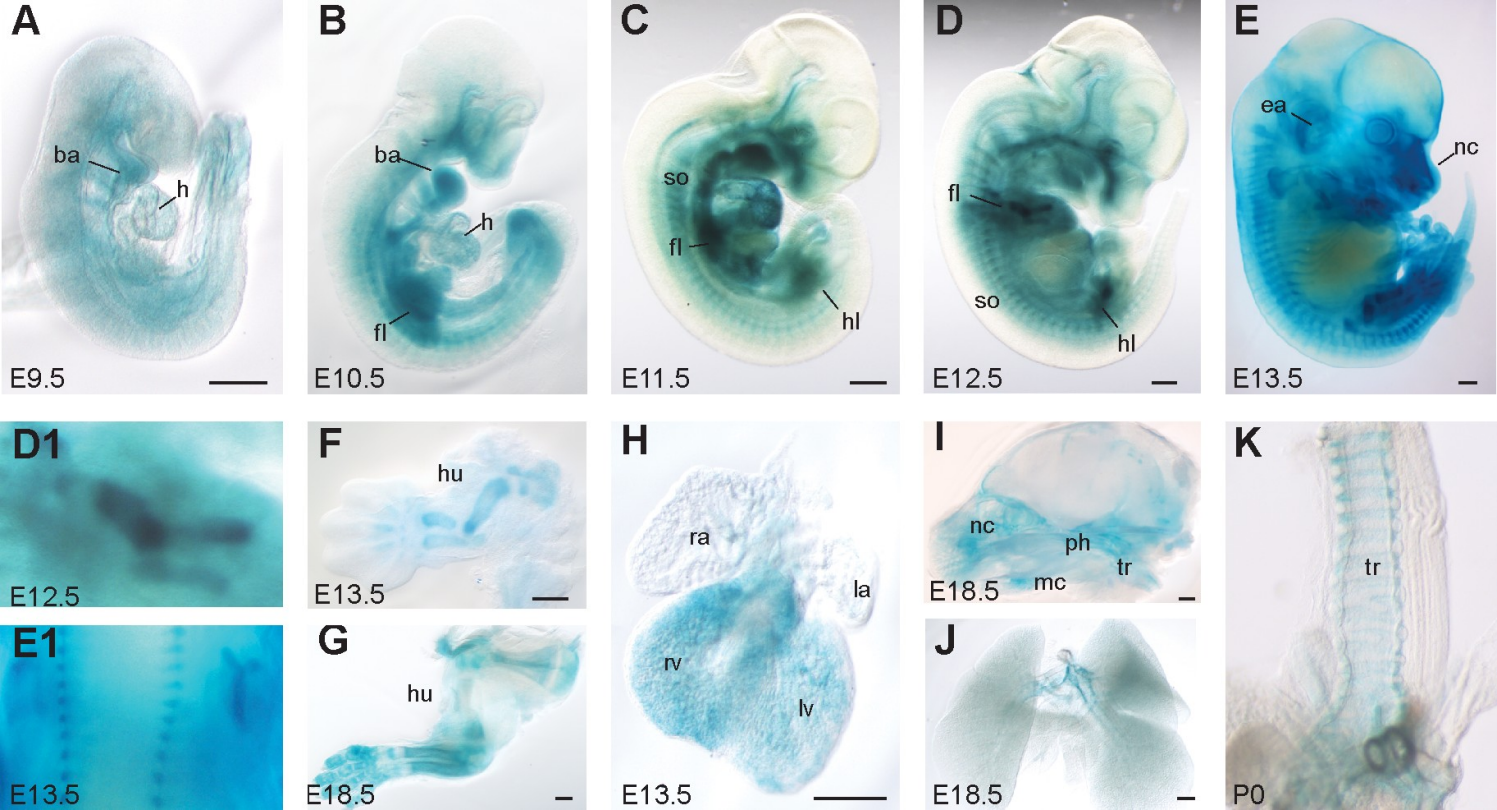
ASAP1 is strongly expressed in the developing skeleton, heart and trachea. X-Gal staining of *Asap1*^*GT*^ embryos at E9.5 (**A**), E10.5 (**B**), E11.5 (**C**), E12.5 (**D**), E13.5 (**E**). Enlarged images of the developing fore limb of an E12.5 embryo (**D1**) and the spine of an E13.5 embyro (**E1**). (**F and G**) Staining of prepared forelimbs of E13.5 and E18.5 embryos. (**H**) β-geo reporter expression in the E13.5 heart. (**I and J**) β-geo reporter expression in the skull and lung at E18.5. **(K)** β-geo reporter expression in the trachea at P0. Scale bars: 0.5 mm. Abbreviations: ba, branchial arch; ea, ear; fl, fore limb; h, heart; hl, hind limb; hu, humerus; la, left atrium; lv, left ventricle; nc, nasal cavity; mc, meckel’s cartilage; ph, pharynx; ra, right atrium; rv, right ventricle; so, somites; tr, trachea.

To characterize the expression pattern of ASAP1 in embryonic and adult tissues, the β-geo reporter integrated into the ASAP1 locus of *Asap1*^*GT*/GT^ mice was used. Homozygous *Asap1*^*GT*/GT^ embryos of different embryonic stages were stained with X-Gal. In E9.5 embryos, the β-geo reporter was widely expressed, including in the heart and branchial arches (Fig 2A). At E10.5 and E11.5, ASAP1 was expressed in the heart, the branchial arches, the limb buds and in the somites (Fig 2B and 2C). In E12.5 embryos, ASAP1 was strongly expressed in the Y-shaped mesenchymal condensations of the developing limbs and in the developing pharynx (Fig 2D and 2D1). From E13.5 to P0, β-geo reporter expression was found in the somites (Fig 2E and 2E1), the developing limbs (Fig 2F and 2G), the nasal capsule, pharynx and cochleae (Fig 2E and 2I), the heart (Fig 2H), and the trachea and bronchi (Fig 2J and 2K).

Expression of ASAP1 in the lung bronchi and heart was also confirmed in adult animals, together with expression in the liver, brain, spleen, skin and testis (S2 Fig). Despite the widespread expression of ASAP1 in many tissues, ASAP1-deficient mice that survived the first day after birth were reproductively viable, had equivalent longevity to their wild-type littermates and did not exhibit any predisposition to develop autochthonous tumors.

ASAP1 has been implicated in angiogenesis and endothelial cell migration [37]. We also found that ASAP1 is expressed in lymphatic vessels (S3 Fig). However, ASAP1-deficient mice did not exhibit obvious defects in the vasculature. Nevertheless, to investigate a possible role for ASAP1 during angiogenesis and/or lymphangiogenesis in more detail, we examined angiogenesis in neonatal retinas from *Asap1*^+/+^ and *Asap1*^*GT*/GT^ mice, and performed *ex vivo* thoracic duct ring assays to investigate possible differences in lymphangiogenesis. Using these assays, no differences in angiogenesis or lymphangiogenesis between the different genotypes were observed (S3 Fig).

### Loss of ASAP1 delays ossification during embryogenesis

**Fig 3:**
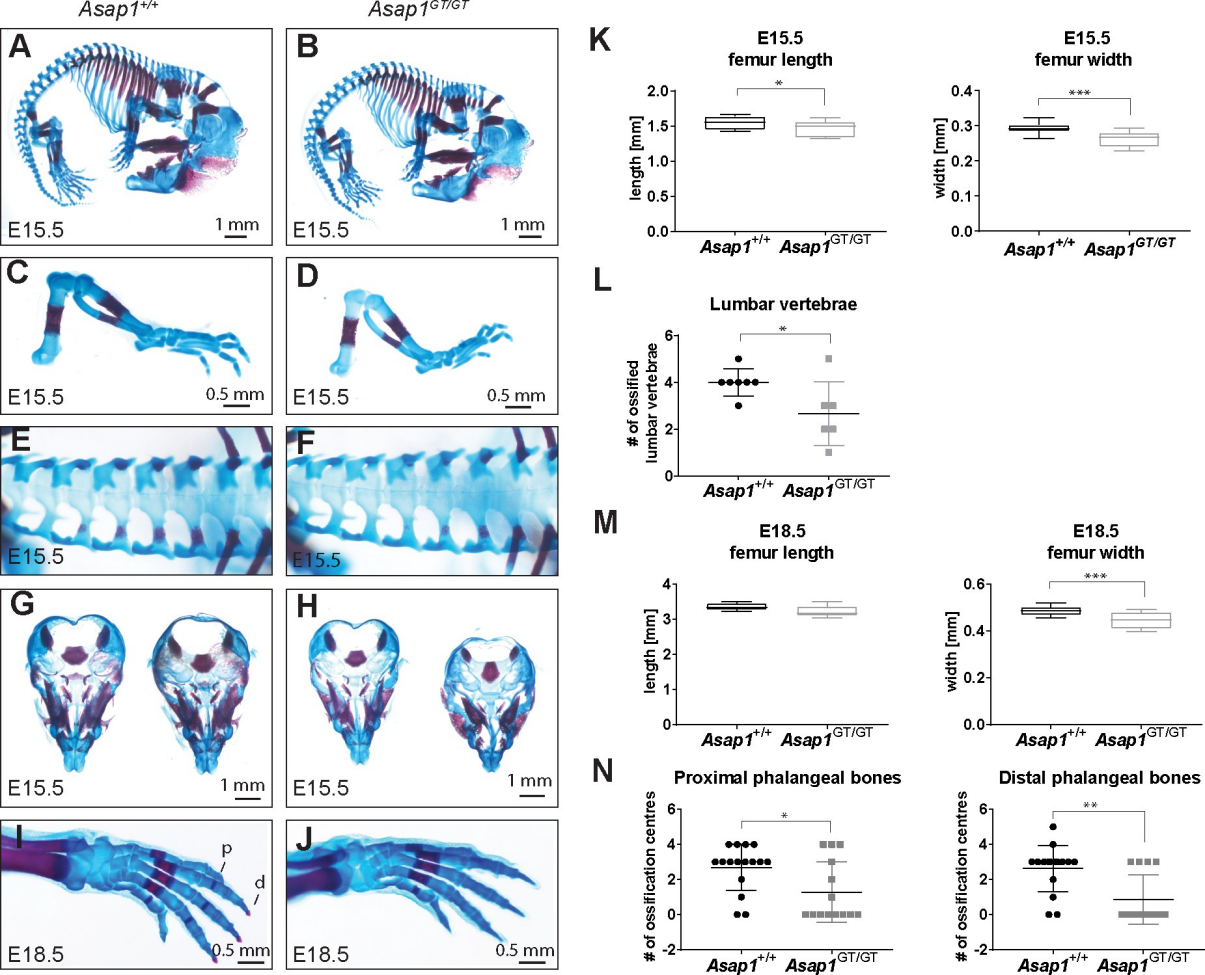
Loss of ASAP1 delays ossification. (**A-H**) Chondroskeletal staining of E15.5 *Asap1*^+/+^ (**A, C, E, G**) and *Asap1*^*GT*/GT^ embryos (**B, D, F, H**), demonstrating reduced whole body size (**A, B**) and reduced size and ossification of hind limbs (**C, D**), lumbar vertebra (**E, F**) and skull (**G, H**) in *Asap1*^*GT*/GT^ embryos. (**I, J**) Chondroskeletal staining of E18.5 embryos, demonstrating reduced ossification of proximal and distal phalangeal bones in *Asap1*^*GT*/GT^ embryos. (**K, L**) Quantification of femur length, width and number of ossified lumbar vertebrae in E15.5 embryos (*Asap1*^+/+^ n = 7, *Asap1*^*GT*/GT^ n = 6). (**M, N**) Quantification of femur length and width (*Asap1*^+/+^ n = 5, *Asap1*^*GT*/GT^ n = 5) and the number of proximal and distal ossification centers in E18.5 embryos (*Asap1*^+/+^ n = 8, *Asap1*^*GT*/GT^ n = 8, left and right). For K and M, the box-whisker plots show the mean values and the 25^th^ and 75^th^ percentiles ± SD. For L and N, graphs show the mean ± SD. Significance was calculated using the Student’s t-test*, p < 0.05, ** p < 0.005, *** p < 0.001. p, proximal; d, distal.

The strong expression of ASAP1 in the cartilage tissue during mouse development and the growth retardation observed at birth prompted us to investigate whether loss of ASAP1 results in defective cartilage or bone formation. Chondroskeletal staining was performed using E15.5 and E18.5 wild-type and *Asap1*^*GT*/GT^ embryos. At E15.5, we found that *Asap1*^*GT*/GT^ embryos were smaller in size, had shorter skulls and that the length and width of the femur was reduced compared to wild-type animals (Fig 3A–3D, 3G, 3H and 3K). Furthermore, ossification of the lumbar vertebrae, the frontal bone of the cranial vault, the premaxilla and the mandible of *Asap1*^*GT*/GT^ embryos was delayed (Fig 3E–3H and 3L). At E18.5, reduced femur width was again observed in *Asap1*^*GT*/GT^ embryos, although the femur length was not significantly different between *Asap1*^+/+^ and *Asap1*^*GT*/GT^ embryos (Fig 3M). Furthermore, the ossification of the proximal and distal phalanges was delayed in *Asap1*^*GT*/GT^ compared to wild-type embryos (Fig 3I, 3J and 3N). Together, these data suggest that loss of ASAP1 delays bone development.

**Fig 4:**
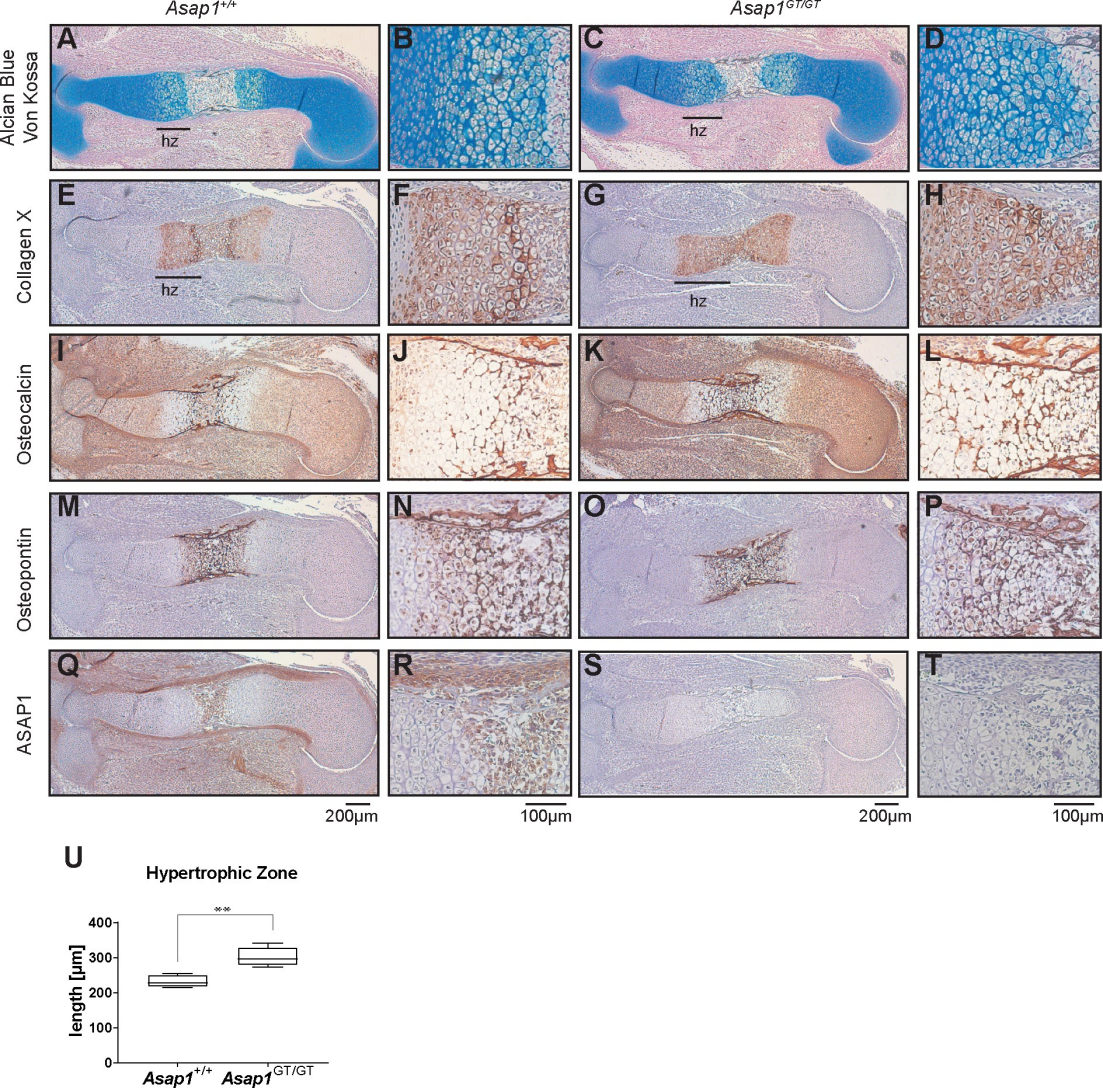
Loss of ASAP1 results in expansion of the hypertrophic zone. (**A-D**) E15.5 *Asap1*^+/+^ and *Asap1*^*GT*/GT^ growth plates of the humerus stained for Alcian Blue / von Kossa in blue and black, respectively. The length of the hypertrophic zone (hz) is demarcated by a black bar. (**E-T**) Immunostaining of E15.5 *Asap1*^+/+^ and *Asap1*^*GT*/GT^ humeral growth plates. Staining of the hypertrophic marker collagen X **(E-H)** and the osteogenic markers osteocalcin (**I-L**) and osteopontin (**M-P**) was detected by Nova Red and counterstained with hematoxilin. ASAP1 immunostaining indicates specific expression of ASAP1 in the perichondrium, in late hypertrophic chondrocytes and in the primary ossification center (**Q-T**). The scale bars shown at the bottom of the panels apply to all pictures above the given scale bar. (**U**) Quantification of the distal hypertrophic zones of the humerus of *Asap1*^+/+^ and *Asap1*^*GT*/GT^ animals (*Asap1*^+/+^ n = 5, *Asap1*^*GT*/GT^ n = 5). Graphs show the mean ± SD. Significance was calculated using the Student’s t-test. **, p <0.005.

To investigate the role of ASAP1 in bone development in more detail, the consequence of loss of ASAP1 on growth plate development in E15.5 embryos was analyzed. Alcian Blue/ von Kossa double staining revealed an increase in the hypertrophic zone in *Asap1*^*GT*/GT^ animals (Fig 4A–4D and 4U). Consistently, the expression area of the hypertrophic marker collagen X and the osteogenic markers osteocalcin and osteopontin was expanded, and the chondro-osseous junction (the transition between chondrocytes and mineralized bone) appeared disorganized, indicative of premature hypertrophy (Fig 4E–4P). Interestingly, ASAP1 was expressed in the perichondrium, in osteoblasts and in terminally differentiated chondrocytes, suggesting a role in late chondrogenesis and the genesis of osteoblasts (Fig 4Q–4T).

### Loss of ASAP1 results in reduced adipogenesis *in vivo*

**Fig 5:**
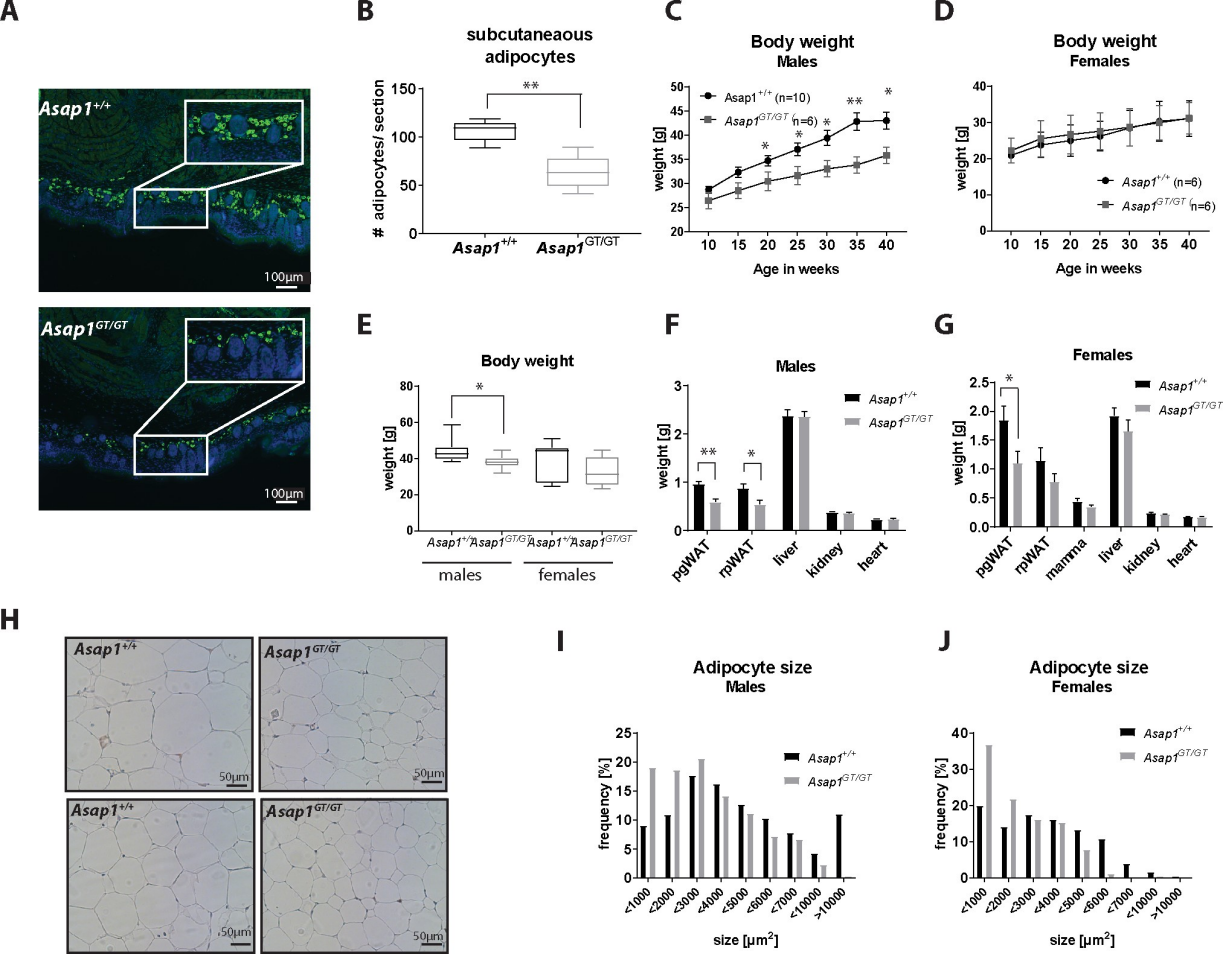
Loss of ASAP1 results in decreased numbers of neonatal subcutaneous adipocytes and reduced body weight. (**A**) Transverse sections at the level of the scapulae of neonatal WT and *Asap1*^*GT*/GT^ embryos were stained for perilipin A (green fluorescence) and DAPI (blue), which demonstrated a reduced number of perilipin A-positive subcutaneous adipocytes in *Asap1*^*GT*/GT^ neonates (P0). (**B**) Quantification of the number of subcutaneous adipocytes. The number of perilipin A-positive cells per picture of three adjacent scapulae sections was counted and averaged. The box-whisker plots show the mean values and the 25^th^ and 75^th^ percentiles for *Asap1*^+/+^ (n = 5) and *Asap1*^*GT*/GT^ embryos (n = 6) ± min to max. (**C**) Body weight (BW) of male WT and *Asap1*^*GT*/GT^ mice at the age of 10 to 40 weeks. Data represents mean ± SEM (*Asap1*^+/+^, n = 10 and *Asap1*^*GT*/GT^, n = 6). (**D**) Body weight (BW) of female WT and *Asap1*^*GT*/GT^ mice at the age of 10 to 40 weeks. Data represents mean ± SD (*Asap1*^+/+^, n = 6 and *Asap1*^*GT*/GT^, n = 6). (**E**) Body weight of male and female WT and *Asap1*^*GT*/GT^ mice at the age of 18 months. The box-whisker plots show the mean values and the 25^th^ and 75^th^ percentiles for *Asap1*^+/+^ (male n = 11, female n = 9) and *Asap1*^*GT*/GT^ (male n = 8, female n = 8) ± min to max. (**F and G**) Weight of perigonadal WAT (pgWAT), retroperitoneal WAT (rpWAT), liver, kidney and heart of WT and *Asap1*^*GT*/GT^ mice at the age of 18 months. (**H**) Representative pictures of male (upper panels) and female (lower panels) pgWAT of aged WT and *Asap1*^*GT*/GT^ mice (age 18 months). (**I**) Distribution of adipocyte size in pgWAT from WT and *Asap1*^*GT*/GT^ male mice (*Asap1*^+/+^ n = 6, *Asap1*^*GT*/GT^ n = 5). (**J**) Distribution of adipocyte size in pgWAT from WT and *Asap1*^*GT*/GT^ female mice (*Asap1*^+/+^ n = 4, *Asap1*^*GT*/GT^ n = 4). Significance was calculated using the Student’s t-test.*, p < 0.05, ** p < 0.005.

*Asap1*^*GT/GT*^ neonates showed reduced body weight compared to wild-type animals (Fig 1F). As ASAP1 overexpression promotes the adipogenic differentiation of immortalized 3T3 fibroblasts through its proline-rich and SH3 domains [38], we investigated whether the decreased body weight of ASAP1 deficient mice is associated with reduced adipocyte development. The development of white adipose tissue occurs largely after birth, but sub-dermal lipid-filled adipocytes can already be detected around birth [9]. We analyzed the subcutaneous region of the scapula for perilipin A-positive cells, and found that *Asap1*^*GT/GT*^ neonates exhibit significantly fewer subcutaneous adipocytes compared to their *Asap1*^+/+^ littermates (Fig 5A and 5B). Furthermore, the body weight of male and female mice was monitored for several months, which revealed that male *Asap1*^*GT/GT*^ mice gained less weight compared to WT littermates, whereas there was no difference in females (Fig 5C and 5D). At the age of 18 months, the bodyweight of male *Asap1*^*GT/GT*^ mice was significantly lower compared to control mice, and there was a similar trend in female *Asap1*^*GT/GT*^ mice (Fig 5E). All mice had smaller perigonadal and retroperitoneal fat depots compared to their WT littermates, while other organs such as liver, kidney or heart were not affected (Fig 5F and 5G). In addition to a decrease in the weight of fat depots, the size of adipocytes in perigonadal WAT was also reduced in *Asap1*^*GT/GT*^ mice (Fig 5H–5J). These observations suggest that *Asap1*^*GT/GT*^ mice have a defect in the generation of adipocytes and the accumulation of lipids, supporting the notion that ASAP1 promotes adipogenesis *in vivo*.

### Loss of ASAP1 interferes with osteo- and adipogenic terminal differentiation

The results above suggest that ASAP1 is involved in the generation of chondrocytes, osteoblasts and adipocytes, differentiated cell lineages that are derived from mesenchymal progenitor cells. Therefore, we investigated the role of ASAP1 during mesenchymal differentiation *in vitro*.

**Fig 6:**
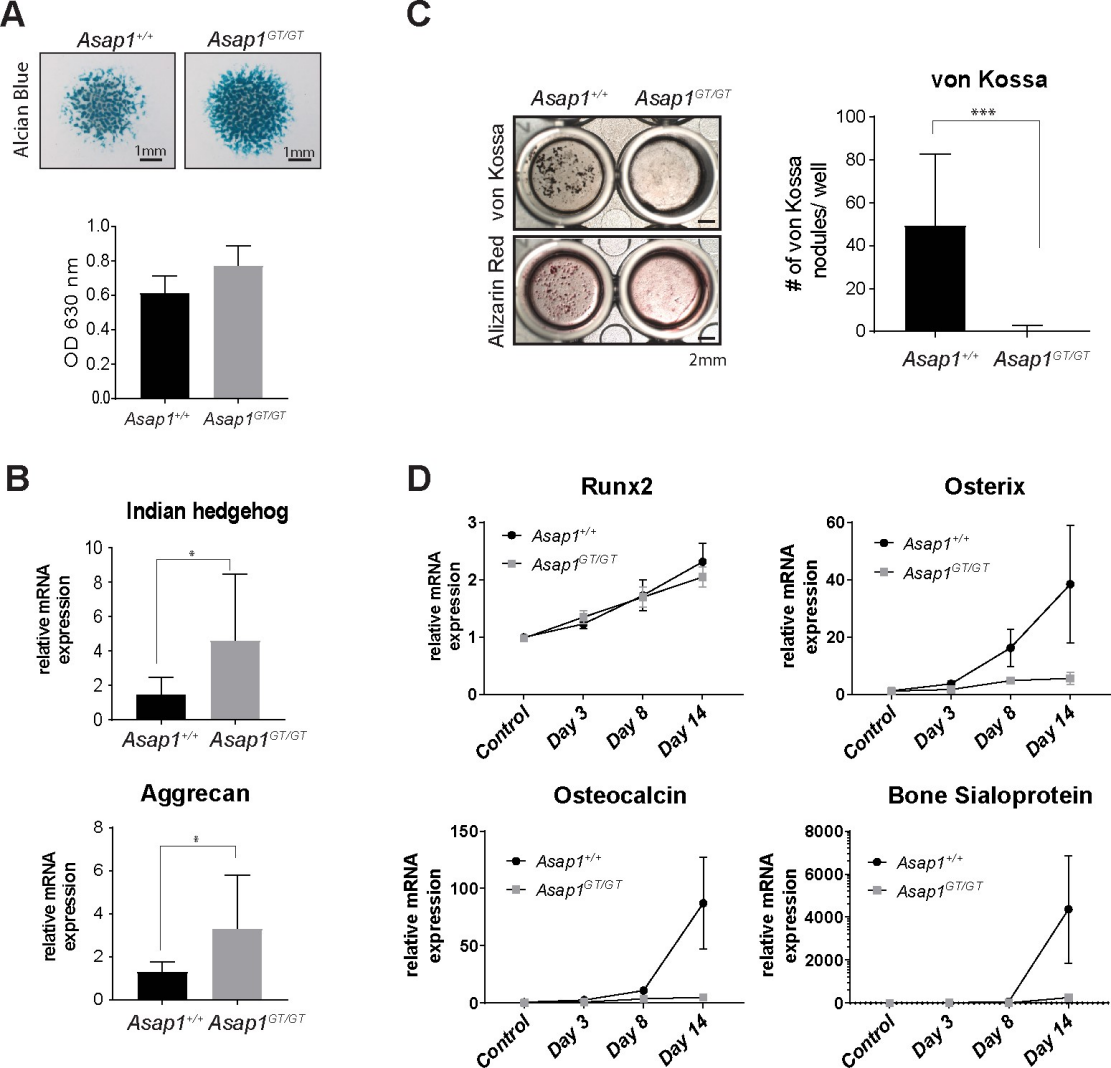
Loss of ASAP1 promotes chondrogenesis but prevents mineralization *in vitro*. (**A**) Limb bud mass cultures of *Asap1*^+/+^ and Asap1^GT/GT^ embryos were stained with Alcian Blue after seven days of differentiation. Two representative pictures per genotype are shown (left panel). Alcian Blue was extracted and the OD measured at 630 nm. The data represent the mean ± SD (*Asap1*^+/+^ n = 18, *Asap1*^*GT*/GT^ n = 18). Scale bars: 1 mm. Significance was calculated using the Student’s t-test (right panel). (**B**) qPCR analysis of limb bud mass cultures after seven days of differentiation. Relative mRNA expression was analyzed for aggrecan and Indian hedgehog, and normalized to RibP0 expression. Data represent the mean ± SD (*Asap1*^+/+^ n = 8, *Asap1*^*GT*/GT^ n = 8). Significance was calculated using the Student’s t-test.* p< 0.05. (**C**) *Asap1*^+/+^and *Asap1*^*GT*/GT^ MEFs were differentiated into the osteogenic lineage and stained for mineral deposition by von Kossa and Alizarin Red. Representative pictures of differentiated *Asap1*^+/+^ and *Asap1*^*GT*/GT^ MEFs are shown (left panel). Quantification of von Kossa positive nodules (right panel). The graph represents the mean ± SD of three biological replicates for three independent MEF cell lines per genotype. Scale bars: 2 mm. Significance was calculated using the Student’s t-test. *** p < 0.0005 (**D**) Late osteogenic markers are not induced in ASAP1-deficient cells. qPCR analysis of differentiating MEFs at day 3, day 8 and day 14 and untreated control cells. Relative mRNA expression was analyzed for Runx2, ALP, osteopontin, osterix, osteocalcin and bone sialoprotein, and normalized to expression of RibP0. Data represent the mean ± SEM (*Asap1*^+/+^, n = 4 and *Asap1*^*GT*/GT^, n = 5).

To investigate the role of ASAP1 during chondrogenic differentiation, limb bud mass cultures from *Asap1*^+/+^ and *Asap1*^*GT*/GT^ E11.5 embryos were stained with Alcian Blue to detect proteoglycan synthesis. *Asap1*^*GT*/GT^ cultures showed a trend towards increased Alcian Blue staining compared to wild-type controls (Fig 6A). Furthermore, transcription of chondrogenic markers such as aggrecan and Indian hedgehog was significantly increased in *Asap1*^*GT*/GT^ compared to wild-type limb bud mass cultures (Fig 6B), suggesting that loss of ASAP1 promotes chondrogenesis, consistent with the finding that loss of ASAP1 results in expansion of the hypertrophic zone.

Next, we differentiated *Asap1*^+/+^ and *Asap1*^*GT*/GT^ primary MEFs into the osteogenic lineage to investigate the regulation of osteogenesis by ASAP1. After 14 days of differentiation-inducing treatment, *Asap1*^+/+^ and *Asap1*^*GT*/GT^ MEFs were assessed for osteogenic differentiation. In *Asap1*^+/+^ cells, von Kossa-positive nodules and Alizarin Red staining could be readily detected (Fig 6C). In contrast, no von Kossa or Alizarin Red staining was observed in *Asap1*^*GT*/GT^ cells. Impaired osteogenic differentiation in *Asap1*^*GT*/GT^ cells was further confirmed by analyzing transcription of the osteogenic transcription factor osterix and the matrix proteins osteocalcin and bone sialoprotein. *Asap1*^+/+^ cells showed a strong increase in expression of these factors, whereas no expression was detected in *Asap1*^*GT*/GT^ cells (Fig 6D). Interestingly, the induction of Runx2 was not affected by the loss of ASAP1, suggesting that part of the osteogenic differentiation program is induced in the absence of ASAP1, but cannot be completed.

**Fig 7:**
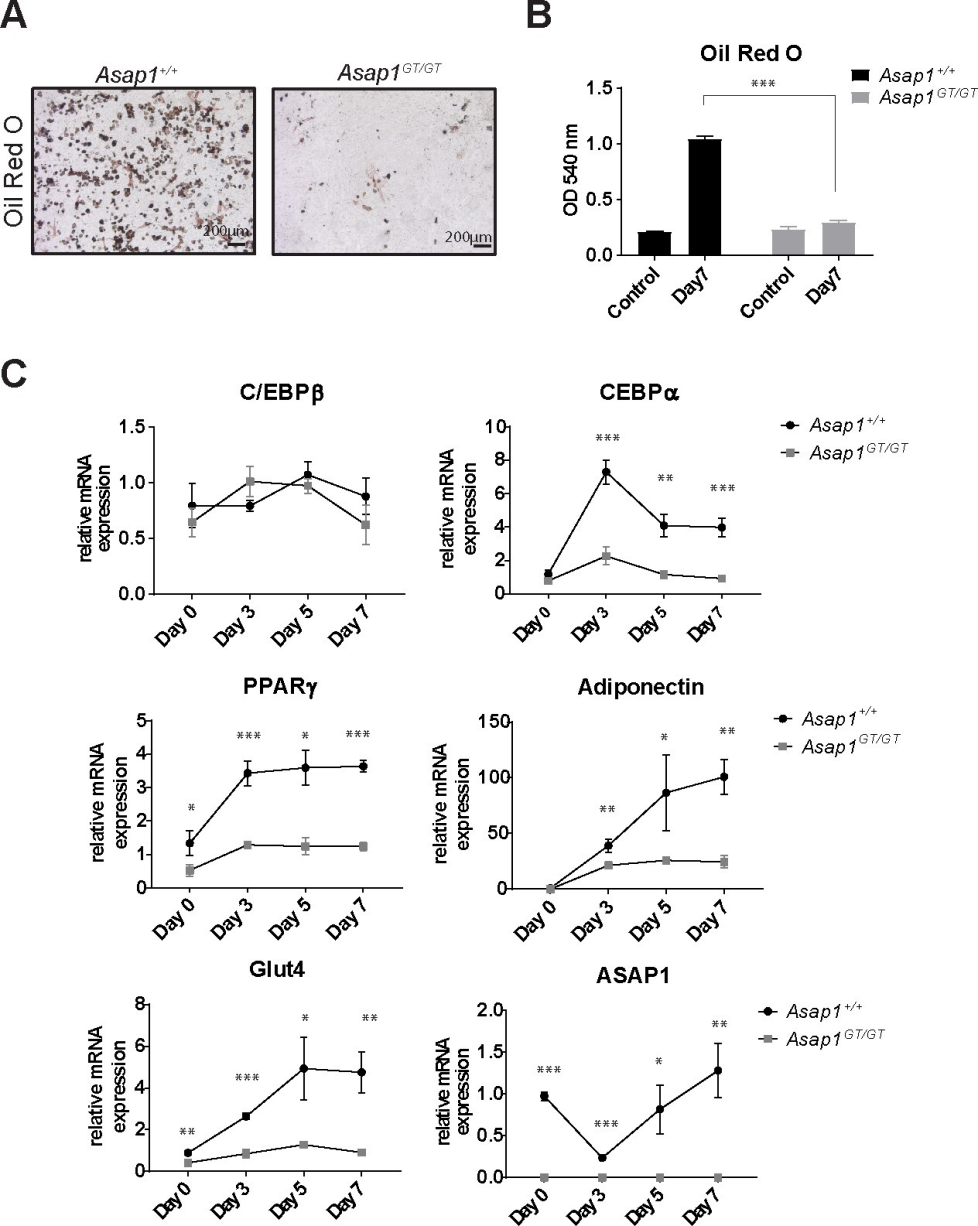
Loss of ASAP1 abrogates adipogenic differentiation *in vitro*. (**A**) *Asap1*^+/+^and *Asap1*^*GT*/GT^ MEFs were induced to differentiate into the adipogenic lineage, and stained for Oil Red O. Representative images of uninduced controls and differentiated cells after 7 days are shown (upper panel). Scale bars: 200 μm. (**B**) Quantification of Oil Red O staining (lower panel). Oil Red O was extracted and the OD measured at 540 nm. The graph represents the mean ± SD of one representative experiment out of three performed, each of which was performed with three biological replicates. Significance was calculated using the Student’s t-test. *** p < 0.0005. (**C**) Adipogenic markers are not induced in differentiated ASAP1-deficient cells. qPCR analysis of differentiating MEFs at day 0, 3 and day 7. Relative mRNA expression was analyzed for C/EBPβ, C/EBPα, PPARγ, adiponectin, Glut4 and ASAP1, and normalized to expression of RibP0. Data represent the mean ± SD (*Asap1*^+/+^, n = 3 and *Asap1*^*GT*/GT^, n = 3). Significance was calculated using the Student’s t-test. *, p < 0.05, ** p < 0.005.

Finally, we investigated whether loss of ASAP1 also impacts on adipogenesis. Primary MEFs were differentiated into the adipogenic lineage by addition of insulin, IBMX, dexamethasone and troglitazone. After 7 days of differentiation, *Asap1*^+/+^ and *Asap1*^*GT/GT*^ cells were stained for Oil Red O, and analyzed for adipogenic gene expression. *Asap1*^+/+^ cells strongly differentiated into adipocytes, as demonstrated by Oil Red O staining (Fig 7A and 7B), began to express the adipogenic marker C/EBPα and PPARγ after 3 days, and showed high expression of PPARγ, adiponectin and Glut4 after 5 and 7 days (Fig 7C). By contrast, differentiated *Asap1*^*GT/GT*^ MEFs stained poorly with Oil Red O. Although C/EBPβ was regulated similarly to *Asap1*^+/+^ cells, the *Asap1*^*GT/GT*^ cells did not exhibit subsequent high induction of PPARγ and C/EBPα, or later expression of the adipogenic markers adiponectin and Glut4 (Fig 7C). Interestingly, ASAP1 expression was strongly suppressed in *Asap1*^+/+^ cells during early adipogenic differentiation, but returned to basal levels after 7 days. These results demonstrate that ASAP1 is required for terminal differentiation of mesenchymal progenitor cells into adipocytes.

### ASAP1 promotes adipogenic differentiation through regulating FAK/Src and Akt signaling

**Fig 8:**
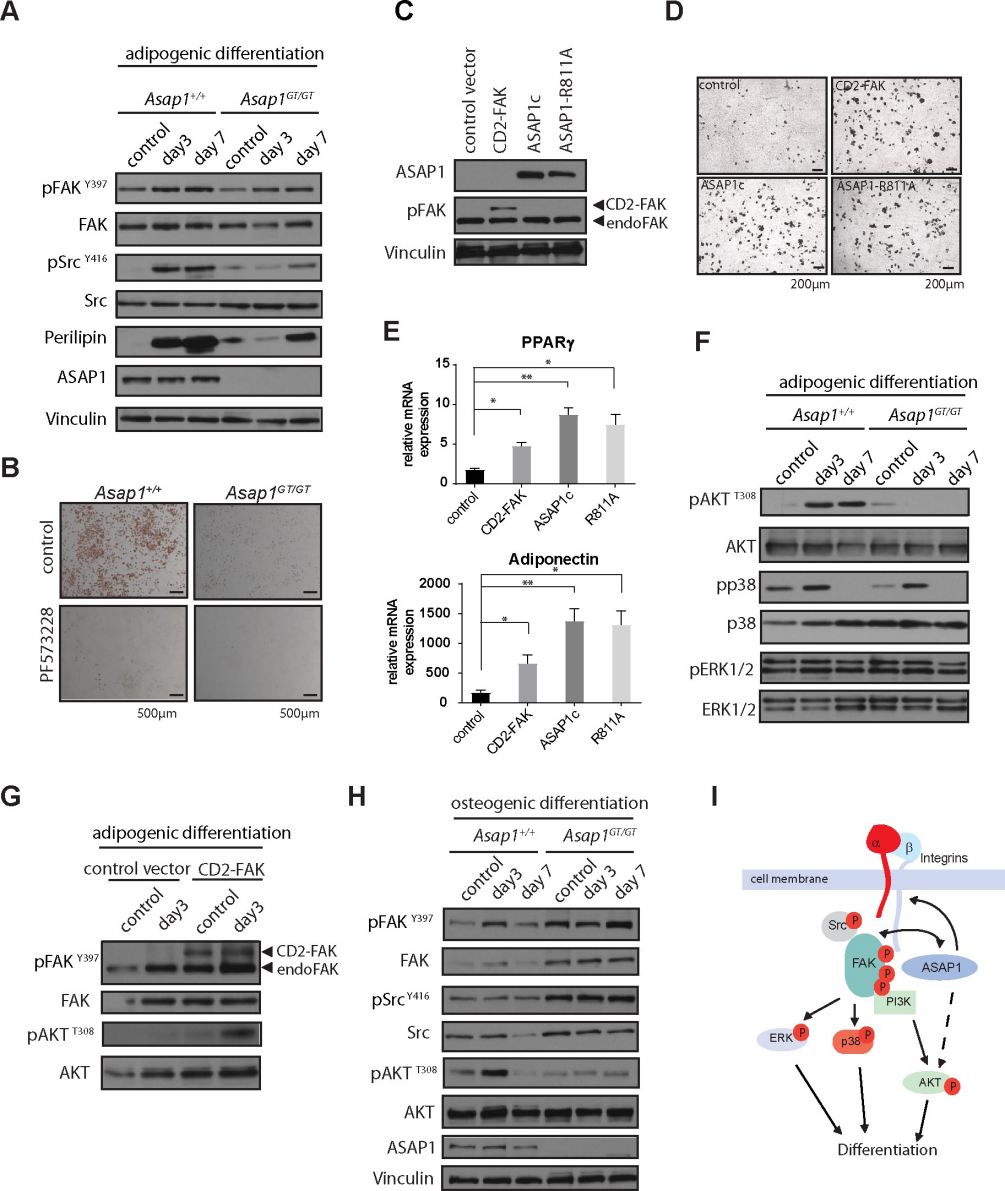
FAK/ Src and Akt signaling is impaired in *Asap1*^GT/GT^ MEFs, which inhibits adipogenic differentiation. (**A**) FAK and Src phosphorylation is impaired when *Asap1*^*GT*/GT^ MEFs are induced to differentiate into the adipogenic lineage. Protein lysates of undifferentiated controls and WT and *Asap1*^*GT*/GT^ MEFs at day 3 and day 7 after induction of differentiation were analyzed by Western blotting. Blots were probed with the antibodies indicated on the left. (**B**) Inhibition of FAK activity by PF-573228 prevents adipogenesis in MEFs. WT and *Asap1*^*GT*/GT^ MEFs were induced to differentiate into the adipogenic lineage with or without 10 μM PF-573228. Scale bars: 500 μm. (**C**) *Asap1*^*GT*/GT^ MEFs were infected with control, CD2-FAK, ASAP1c or ASAP1-R811A retroviruses. Protein lysates of infected MEFs were analyzed by Western blotting. Blots were probed with the antibodies indicated on the left. (**D**) Enforced FAK activity in *Asap1*^*GT*/GT^ MEFs rescues the induction of adipogenesis. Control *Asap1*^*GT*/GT^ MEFs and *Asap1*^*GT*/GT^ MEFs ectopically expressing CD2-FAK, ASAP1c or ASAP1-R811A were differentiated into the adipogenic lineage, and after seven days of differentiation they were stained with Oil Red O. Scale bars: 200 μm. (**E**) Control *Asap1*^*GT*/GT^ MEFs and *Asap1*^*GT*/GT^ MEFs ectopically expressing CD2-FAK, ASAP1c or ASAP1-R811A were differentiated into the adipogenic lineage. At adipogenic day 7, relative mRNA expression was analyzed for PPARγ and adiponectin, with normalization relative to the expression of RibP0. Data represent the mean ± SD. Significance was calculated using the Student’s t-test. *, p < 0.05, ** p < 0.01. (**F**) AKT signaling is abrogated when *Asap1*^*GT*/GT^ MEFs are induced to differentiate. Protein lysates of undifferentiated controls and WT and *Asap1*^*GT*/GT^ MEFs at day 3 and day 7 after induction of differentiation were analyzed by Western blotting. Blots were probed with the antibodies indicated on the left. (**G**) Constitutively active FAK induces AKT phosphorylation when *Asap1*^*GT*/GT^ MEFs are induced to differentiate. Protein lysates of undifferentiated controls and *Asap1*^*GT*/GT^ MEFs expressing empty control vector or CD2-FAK at day 3 after induction of adipogenic differentiation were analyzed by Western blotting. Blots were probed with the antibodies indicated on the left. (**H**) Src and AKT signaling is dysregulated when *Asap1*^*GT*/GT^ MEFs are induced to differentiate into the osteogenic lineage. Protein lysates of undifferentiated controls and WT and *Asap1*^*GT*/GT^ MEFs at day 3 and day 7 after induction of osteogenic differentiation were analyzed by Western blotting. Blots were probed with the antibodies indicated on the left. (**I**) Schematic model of ASAP1-dependent signaling during the differentiation of mesenchymal progenitor cells, proposed on the basis of the results in this paper and other observations in the literature. FAK activation by integrins leads to auto-phosphorylation of T^397^, a process regulated by ASAP1 through regulation of integrin recycling. T^397^ phosphorylation enables recruitment and activation of Src, leading to full activation of FAK, and binding of the PI3K regulatory subunit to FAK. ASAP1 regulates FAK/Src and AKT phosphorylation. Activation of the FAK/Src complex results in phosphorylation of down-stream signaling pathways including ERK, p38 and AKT, which in turn determine the differentiation fate of mesenchymal progenitor cells.

To understand at the mechanistic level how loss of ASAP1 suppresses adipogenic differentiation *in vivo* and *in vitro*, we next investigated the impact of ASAP1 deficiency on key regulatory signaling proteins. As ASAP1 interacts with FAK and Src via its proline-rich and SH3 domains [23, 26], and both molecules are functionally involved in adipogenic differentiation [14, 15], we hypothesized that dysregulated FAK/Src signaling may contribute to the phenotype observed in *Asap1*^*GT/GT*^ MEFs. Consistent with this notion, we found that phosphorylation of FAK was reduced in ASAP1-deficient MEFs after induction of adipogenic differentiation compared to wild-type controls (Fig 8A). Furthermore, phosphorylation of Src was also reduced (Fig 8A). These results indicate that FAK/ Src signaling is hampered in *Asap1*^*GT/GT*^ MEFs, consistent with incomplete adipogenic differentiation.

Next, we used loss and gain of function approaches to demonstrate a mechanistic role for FAK in ASAP1-dependent adipogenic differentiation. Treatment of MEFs with the FAK inhibitor PF-573228 completely abrogated adipogenesis in WT MEFs (Fig 8B). Ectopic expression of a constitutive active form of FAK (CD2-FAK) in *Asap1*^*GT/GT*^ MEFs rescued the induction of adipogenic differentiation almost as effectively as re-introduction of ASAP1 into the cells (Fig 8C–8E). Together, these data suggest that FAK is involved functionally in ASAP1-dependent adipogenic differentiation. Interestingly, mutation of the proline-rich domain P1 of ASAP1 (ASAP1-R811A), which decreases binding of ASAP1 to Src SH3 domain proteins including Src, did not impair the ability of ASAP1 to restore adipogenic differentiation in *Asap1*^*GT/GT*^ MEFs (Fig 8C–8E).

ERK, p38 and AKT are signal transduction components down-stream of FAK that have been implicated in regulating adipogenesis [14, 39, 40]. We therefore hypothesized that activation of these components may be impaired in *Asap1*^*GT/GT*^ MEFs after induction of adipogenic differentiation. Consistent with this notion, induction of adipogenic differentiation in WT MEFs strongly induced activation of AKT and p38 through phosphorylation of key residues (Fig 8F). By contrast, activation of AKT was completely abrogated, and activation of p38 was significantly impaired upon induction of adipogenic differentiation in *Asap1*^*GT/GT*^ MEFs, while phosphorylation of ERK was not significantly affected (Fig 8F). As AKT signaling is essential for insulin-induced adipogenic differentiation [41], these results suggest that the ability of ASAP1 to promote adipogenic differentiation may also require AKT activation. We therefore investigated whether constitutively active FAK activates AKT signaling in *Asap1*^*GT/GT*^ MEFs to rescue the adipogenic phenotype. Indeed, phosphorylation of AKT T308 was induced when *Asap1*^*GT/GT*^ MEFs expressing constitutively active FAK were differentiated into the adipogenic lineage (Fig 8G).

Given the role of ASAP1 in regulating cytoskeletal dynamics and adhesion, we analyzed focal adhesions in WT and *Asap1*^*GT*/GT^ MEFs before and during induction of adipogenesis. No obvious differences were observed in the distribution, size or number of focal adhesions in WT and *Asap1*^*GT/GT*^ MEFs (S4 Fig). We also did not observe differences in cell adhesion on a variety of substrates when WT and *Asap1*^*GT/GT*^ MEFs were compared (S4 Fig). Together, these results suggest that the FAK/Src-PI3K/AKT signaling pathway is defective in *Asap1*^*GT/GT*^ MEFs, which leads to impaired adipogenic differentiation, but does not impact on focal adhesion formation or cell adhesion.

### Loss of ASAP1 deregulates Src and AKT signaling during osteogenesis

FAK/Src and AKT also play pivotal roles during osteogenesis [16, 17, 42]. We therefore investigated FAK/Src and AKT signaling upon induction of osteogenic differentiation in *Asap1*^*GT/GT*^ MEFs. FAK protein levels were slightly increased in *Asap1*^*GT/GT*^ MEFs compared to *Asap1*^+/+^ MEFs under osteogenesis-inducing conditions, but the degree of FAK phosphorylation appeared similar in both genotypes (Fig 8H). However, compared to *Asap1*^+/+^ MEFs, phosphorylation of Src was strongly increased in *Asap1*^*GT/GT*^ MEFs upon osteogenic stimuli (Fig 8H). As Src activation prevents osteogenesis [17], this may explain why *Asap1*^*GT/GT*^ MEFs exhibit an impaired ability to differentiate into the osteogenic lineage. Furthermore, AKT phosphorylation was strongly induced in WT MEFs upon induction of osteogenic differentiation whereas it was completely attenuated in *Asap1*^*GT/GT*^ MEFs. These results suggest that dysregulation of FAK/Src-PI3K/AKT signaling in *Asap1*^*GT/GT*^ MEFs may also contribute to diminished osteogenic differentiation.

## Discussion

In this study, we found that ASAP1 plays an essential role in mesenchymal differentiation *in vivo* and *in vitro*. ASAP1 deficiency impaired embryonic growth, delayed ossification, attenuated adipocyte development, and reduced body fat formation. Consistently, mesenchymal progenitor cells deficient in ASAP1 were seriously hampered in their ability to differentiate into the osteogenic and adipogenic lineages. Impaired adipogenic differentiation in *Asap1*^*GT*/GT^ MEFs was rescued by FAK activation, and defective adipogenic and osteogenic differentiation were both associated with dysregulated Src and AKT signaling. These data demonstrate new functional roles for ASAP1 in the differentiation of mesenchymal progenitor cells.

Although ASAP1 deficiency did not impair adult survival or aging, disruption of ASAP1 resulted in around 50% perinatal lethality. Surviving *Asap1*^*GT*/GT^ neonates were growth-retarded and exhibited breathing difficulties at birth. It is therefore conceivable that problems with impaired lung development and inflation at birth, or transient developmental deficits in other components of the respiratory system may contribute to the partial lethal phenotype of *Asap1*^*GT*/GT^ mice.

Several observations implicate ASAP1 in bone development. First, ASAP1 was strongly expressed in the mesenchymal condensations of the developing limbs, and at E15.5, expression was observed in the perichondrium, mature hypertrophic chondrocytes and in cells of the primary spongiosa. Second, delayed ossification of the limbs, vertebrae and of the skull was observed in ASAP1-deficient animals, indicating that ASAP1 plays a general role in endochondral and intramembranous ossification. Third, loss of ASAP1 resulted in an increase in the hypertrophic zone in developing femurs, indicating that ASAP1 may prevent premature hypertrophy. As ASAP1 is not expressed in pre-hypertrophic or hypertrophic chondrocytes, it is conceivable that loss of ASAP1 can influence chondrogenesis by deregulating the tight interaction between the growth plate and perichondrium. In addition, ASAP1 deficiency promoted chondrogenic differentiation and prevented osteogenic differentiation of mesenchymal progenitors *in vitro*. Taken together, our results strongly suggest that ASAP1 is necessary for osteogenic differentiation of mesenchymal progenitor cells, and that loss of ASAP1 expression therefore leads to defects in bone development.

Our data also implicate ASAP1 in regulating adipogenic differentiation. The reduced number of lipid-filled subcutaneous adipocytes in *Asap1*^*GT*/GT^ neonates and the smaller fat depots and reduced size of adipocytes in older *Asap1*^*GT*/GT^ mice compared to their wild-type littermates is consistent with the impaired ability of *Asap1*^*GT*/GT^ MEFs to undergo adipogenic differentiation that we observed in vitro. Mechanistically, we found that adipogenic lineage commitment is not prevented by the loss of ASAP1, as C/EBPβ is not differentially regulated upon adipogenic stimulation. However, expression of the key adipogenic regulators PPARγ and C/EBPα, and late adipogenic genes such as adiponectin and Glut4 were not efficiently induced, suggesting that loss of ASAP1 impedes terminal differentiation.

During the first 40 weeks of life, we observed a difference in male and female weight development. Male *Asap1*^*GT*/GT^ mice were leaner and did not gain as much weight as their WT littermates, while there was no significant difference between female mice. However, when WT and *Asap1*^*GT*/GT^ mice were aged to 18 months, we also observed reduced fat depot weight and adipocyte size in female *Asap1*^*GT*/GT^ mice. Several observations suggest that estrogens may underlie the differences in weight development in female and male *Asap1*^*GT/GT*^ mice compared to wild-type littermates. Male mice are generally more susceptible to obesity and related diseases, and estrogens are thought to protect against the development of obesity and metabolic syndromes [43, 44]. This is reflected by the increased prevalence of insulin resistance and obesity in post-menopausal women. Consistently, ovariectomized mice mimic male mice in their susceptibility to weight gain [44]. Increased estrogen levels may therefore explain why the effect of ASAP1 deficiency on bodyweight and adipogenesis is partially concealed in female *Asap1*^*GT/GT*^ mice.

At the molecular level, ASAP1 physically interacts with FAK and Src [23, 26], two proteins that regulate adipogenic, osteogenic and chondrogenic differentiation [14–19]. We found that loss of ASAP1 impedes activating phosphorylation of FAK and c-Src during adipogenic differentiation of MEFs. A constitutively active form of FAK at least partially rescued the defects in adipogenesis seen in *Asap1*^*GT*/GT^ MEFs, indicating that loss of ASAP1 impairs adipogenesis through defective activation of FAK/Src signaling. ASAP1 has previously been thought to be downstream of FAK and Src signaling [23, 26], but our results indicate that ASAP1 can also affect the activity of both of these proteins. Activation of integrins promotes auto-phosphorylation of FAK, and subsequently the formation of an activated FAK/Src complex [20]. As ASAP1 regulates the recycling of β1 integrins [31], we speculate that loss of ASAP1 may impede activation of FAK/ Src signaling through the regulation of integrin recycling. Furthermore, ASAP1 binds to FAK via its SH3 domain, but binds to Src via its proline-rich domain [23, 26]. We found that mutation of the proline-rich domain of ASAP1 (ASAP1-R811A) did not impair the ability of ASAP1 to rescue adipogenic differentiation in ASAP1-deficient MEFs, which would suggest that direct binding of ASAP1 to Src is not required for ASAP1-mediated adipogenic differentiation.

Analysis of signaling pathways downstream of FAK also revealed that activation of the PI3K-activated serine/threonine kinase AKT was severely hampered in ASAP1-deficient MEFs. PI3K/AKT signaling is a critical component in insulin-induced adipogenesis [41]. Furthermore, deletion of AKT in mice results in severe growth retardation, delayed bone development and impaired adipogenesis [39], reminiscent of the *Asap1*^*GT*/GT^ phenotype we report here. The impaired adipogenic potential of AKT-deficient cells results from an inability to induce PPARγ expression [39]. Thus, the defects in adipogenesis seen in *Asap1*^*GT*/GT^ mice are consistent with impaired activation of down-stream targets of FAK and AKT in *Asap1*^*GT*/GT^ MEFs.

FAK/Src and AKT signaling are also implicated in osteogenesis [16, 17, 42]. Although no apparent differences in FAK phosphorylation were observed between in *Asap1*^*GT*/GT^ and *Asap1*^*+*/+^ MEFs undergoing osteogenic differentiation, *Asap1*^*GT*/GT^ MEFs exhibited markedly increased Src phosphorylation, and attenuated AKT activation. In contrast to the situation in adipogenesis, Src activation inhibits osteogenesis [17, 45]. Therefore, the increased phosphorylation of Src Y416 seen in *Asap1*^*GT*/GT^ MEFs could account for the delayed osteogenesis in *Asap1*^*GT*/GT^ mice. Furthermore, inhibition of AKT prevents osteogenesis, consistent with the attenuated osteogenic differentiation and impaired activation of AKT that we observed in *Asap1*^*GT*/GT^ MEFs [42]. Again, these observations are consistent with the notion that dysregulated FAK/Src and AKT signaling in ASAP1-deficient MEFs contributes to defective osteogenic differentiation.

In contrast to its role in osteogenic and adipogenic differentiation, loss of ASAP1 fostered chondrogenesis. This effect may also be due to dysregulated FAK/Src and AKT signaling. Loss of FAK activity increases chondrogenic differentiation, and inhibition of FAK prevents dedifferentiation of chondrocytes, indicating that FAK negatively regulates chondrogenic differentiation [18, 19]. PI3K/AKT signaling is also activated in proliferating chondrocytes and down-regulated as they terminally differentiate [46]. Therefore, impaired AKT signaling may also contribute to increased chondrogenesis upon loss of ASAP1. Taken together, our results suggest that ASAP1 represents a regulatory hub that coordinates FAK/Src and PI3K/AKT signaling, and thereby regulates the differentiation of mesenchymal progenitor cells (Fig 8I).

ASAP1 has been functionally implicated in the regulation of cell motility *in vitro* due to its ability to coordinate dynamic remodeling of the cytoskeleton, in particular the formation of structures that are required for cell migration such as focal adhesions, podosomes and invadopodia [47]. Key ASAP1 interaction partners that mediate these cytoskeletal changes include FAK and Src [26, 28–30]. AKT, whose activity is deregulated by loss of ASAP1, is also an important regulator of cell motility that acts through cytoskeletal reorganization [48]. Re-arrangement of the actin cytoskeleton plays a pivotal role during mesenchymal progenitor cell differentiation [49–51], and our data show that FAK, Src and AKT activity is deregulated by loss of ASAP1 during adipogenesis and osteogenesis. Although we did not observe any differences in focal adhesion formation when WT and ASAP1-deficient MEFs were compared, there are clear common links at the molecular level between the roles of ASAP1 in cell migration on the one hand, and in regulating the differentiation of mesenchymal progenitor cells on the other.

To our knowledge, this is the first report of an involvement of ASAP1 in regulating AKT signaling. Mechanistically, ASAP1 could affect PI3K/AKT signaling by regulating FAK/Src signaling, as the regulatory subunit of PI3K directly interacts with phosphorylated FAK [22]. However, it is also possible that ASAP1 regulates AKT activity by other means. We note, for example, that Arf1 regulates AKT activation upon growth receptor activation, and that ASAP1 regulates Arf1 through its ArfGAP activity [30, 52]. Thus, ASAP1 deficiency may also impact on AKT signaling through impaired Arf1 activity.

Regulation of AKT activity by ASAP1 may be relevant for cancer progression. ASAP1 is frequently amplified and overexpressed in a variety of cancers, which correlates with metastasis and poor survival [25, 32–34]. As the PI3K/AKT pathway is one of the most commonly dysregulated pathways in cancer [53], future work will investigate whether ASAP1 overexpression can activate AKT signaling in cancer cells, and whether this impacts on the treatment of tumor patients with amplified or overexpressed ASAP1.

## Materials and methods

### Ethics statement

All mice were maintained under conventional housing conditions in accordance with German government and institute guidelines and regulations. Permission was granted by the Regierungspräsidium Karlsruhe for the experiments in this study (Permit Number: 35–9185.81/G-299/16). Mice were sacrificed by cervical dislocation. Embryos were sacrificed by decapitation or immersion in liquid nitrogen.

### Mice

A chimeric mouse (B6; 129P2-*Asap1*^*GT(RRS873)Byg*/Mmucd^) with a gene-trap targeted ASAP1 allele (hereafter referred to as *Asap1*^*GT*^) was obtained from the Mutant Mouse Resource Research Center repository (University of California, Davis, CA, USA) [36] and back-crossed with FVB wild-type mice for at least eight generations to obtain a congenic line on the FBV background. Heterozygous progeny were intercrossed to obtain *Asap1*^+/+^, *Asap1*^+/GT^ and *Asap1*^*GT*/GT^ embryos and neonates for analysis. For embryo analysis, heterozygous *Asap1*^+/GT^ mice were time-mated, and pregnant mice were sacrificed to obtain embryos at the indicated time points. Clearly dead or resorbed embryos were excluded from analysis.

### Whole genome sequencing

A DNA sequencing library from an *Asap1*^*GT*^mouse was produced from 1 μg of genomic DNA following the recommendations of the TruSeq DNA protocol (Illumina, San Diego, CA, USA). Briefly, integrity of the extracted DNA (~100ng) was confirmed by electrophoresis on a 1% agarose gel. DNA was sheared to 200 bp fragments by sonication with a Covaris S220 instrument using the following settings: peak incidence power 175 W, duty factor 10%, cycle per burst 200, time 430 seconds. Fragmented genomic DNA was processed for end-repair, A-tailing, ligation of adapters and enrichment by 11 cycles of PCR following Illumina recommendations. The quality and quantity of the sequencing library was determined on a Bioanalyzer 2100 (DNA1000 chips, Agilent, Santa Clara, CA, USA). Paired-end sequencing (2x50bp) of the library adjusted to 8pM was performed on one lane with TruSeq PE Cluster KIT v3 –cBot–HS and TruSeq SBS KIT v3 –HS kits on the Hiseq1000 (Illumina) platform. Cluster detection and base calling were performed using RTAv1.13 and the quality of reads was assessed with CASAVA v1.8.1 software (Illumina). The sequencing resulted in 120 million 50 nt-long reads (6Gbases), with a mean Phred quality score > 37 for both reads.

To identify the integration site of the GeneTrap vector, we aligned all sequencing reads against the mouse genome (GRCm38/mm10 assembly) with the Burrows-Wheeler aligner BWA 0.6.1 in paired- end mode [54]. Resulting files (BAM format) were merged, filtered out for duplicated pairs and sorted by chromosome coordinates with picard-tools-1.43 (http://picard.sourceforge.net). Visual inspection of the aligned sequences on the mouse genome with IGV2.0 [55] led to the detection of a region of 7.7 kb without mapped reads centered on the ASAP1 exon 3 at the coordinates chr15:64,308,444–64,316,208. Further inspection of read pairs at the border of this region confirmed the presence of chimeric fragments with identity to the targeting vector pGToLxf. This information was used to design a PCR-based genotyping strategy.

### Genotyping the *Asap1*^*GT*^ allele

Genotyping was performed by a three primer PCR-based protocol using genomic DNA prepared either from tail clips of pups or yolk sacks from embryos. The following primers were used: *Asap1*_for 5’-GCATCGCCTGTCATCCTACA-3’, *Asap1*_rev 5’-CTACTTACTCCTACATCTGAGATCC-3’ and *Gene-trap*_for 5’-TCGATGTAACCCACTCGTGC-3’ (see S1 Fig).

### Whole mount lac Z staining

Whole mount *lacZ* staining was performed on embryos of different stages. Dissected embryos were fixed in 4% PFA for 1-2h depending on the developmental age. Fixed embryos were pre-incubated in rinse buffer (PBS, 0.1% Tween 20) and stained overnight in staining solution containing 5 mM K_3_Fe(Cn)_6_, 5 mM K_4_Fe(Cn)_6_ and 1 mg/ml X-Gal. After staining, embryos were washed, post-fixed and cleared in a serial dilution of 20%, 50% and 75% glycerol in 1% KOH. For analysis, stained embryos were submerged in 100% glycerol and pictures were taken with a Leica stereomicroscope (Leica, Wetzlar, Germany).

### Chondroskeletal staining

Alcian Blue and Alizarin Red staining of skeletons was performed as previously published [56]. In brief, E18.5 embryos were de-skinned and eviscerated, while E15.5 embryos were eviscerated only, then fixed in 100% EtOH. After 48h in acetone, embryos were stained overnight at 37°C in 0.005% Alizarin Red (Sigma, St. Louis, MO, USA), 0.015% Alcian Blue (Sigma), and 5% acetic acid in 70% EtOH. Skeletons were cleared in 1% KOH overnight and transferred stepwise into increasing concentrations of glycerol (20%, 50%, 75% in 1% KOH). Pictures of skeletons were taken in 100% glycerol with a Leica stereomicroscope.

### Histological analysis

Limbs and the scapular region from embryos and neonates were fixed in formalin, dehydrated and embedded in paraffin. For immunohistochemical analysis, limbs were decalcified in 15% EDTA for 72h, then 4 μm-thick sections were obtained. Dewaxed and re-hydrated sections of non-decalcified limbs were stained in 1% Alcian Blue in 3% acetic acid or von Kossa stain and counterstained with nuclear fast red. For immunohistochemical analysis, antigen retrieval was performed using proteinase K for osteocalcin, or citrate buffer (DAKO, Santa Clara, CA, USA) for all other antibodies. The following antibodies were used: ASAP1 (#NBP2-48909, Novus biological, Littleton, CO, USA), collagen X (#orb 10444, Biorbyt, San Francisco, CA, USA), osteocalcin (#7060–1815,Biorad, Hercules, CA, USA) osteopontin (#AF808, R&D, Minneapolis, MN, USA) and perilipin A (#P1998, Sigma). Staining was detected by ABC Kit and visualized with Nova Red (Vectorlabs, Burlingame, CA, USA). For immunofluorescence staining, a secondary antibody conjugated with Alexa 488 (Invitrogen, Carlsbad, CA, USA) was used followed by counterstaining with diamidinophenylindole (DAPI). Images were taken using a Zeiss Axio Imager (Zeiss, Oberkochen, Germany).

### Culture and differentiation of mouse embryonic fibroblasts

Primary MEFs were isolated from E13.5 embryos. The head and organs were removed, then embryos were minced and incubated in trypsin overnight at 4°C. The next day, the trypsin was removed, and the tissue was incubated for 15–20 min at 37°C. Then, cells were resuspended in DMEM (Life Technologies, Carlsbad, CA, USA) supplemented with 10% FCS (Sigma), 1% Pen/Strep (Life Technologies) and 2 mM L-glutamine (Life Technologies), and plated. Yolk sacs were used for genotyping. Differentiation experiments were performed with MEFs of passage 2–4. For osteogenic differentiation, cells were plated at 2 x 10^4^ cells/cm^2^ and treated with 50 μg/ml ascorbic acid (#49752, Sigma), 10 Mm β-glycerophosphate (G9422, Sigma) and 200 ng/ml BMP2 (#355-BM, R&D). Cells were incubated with this differentiation cocktail over a period of 14 days with medium changes every 3–4 days. RNA was harvested at the indicated time points. After 14 days of differentiation, cells were fixed in formalin prior to staining with von Kossa stain or Alizarin Red. For adipogenic differentiation, cells were plated at high density (3.5 x 10^4^ cells/ cm^2^) and grown to post-confluency. After seven days, cells were induced to differentiate with 5 μg/ml insulin, 0.5 μM IBMX, 1 μM dexamethasone and 10 μM troglitazone (all from Sigma) for three days. Then, cells were incubated with maintenance medium containing 5 μg/ml insulin and 10 μM troglitazone for two days. Medium was changed to normal growth medium for two additional days. RNA was harvested at the indicated time points, or cells were stained with Oil Red O.

### Limb bud mass culture

Limb buds were dissected from E11.5 embryos and digested in 0.1% collagenase IV and 0.025% trypsin for 30 min at 37°C. Single cells were counted and plated in 20 μl drops at 10^7^cells/ml. After 1h, DMEM F12 medium containing 10% FCS, 50 μg/ml ascorbic acid and 10 mM β-glycerophosphate was added, and cells were cultured for 7 days.

### Histological staining of cells

Mineral deposition was evaluated by von Kossa and Alizarin Red staining. For von Kossa staining, formalin-fixed cells were incubated with 5% AgNO_3_ for 15 minutes followed by 5 minutes of 1% pyrogallol. After fixation with 5% sodium thiosulfate solution, cells were rinsed with H_2_O and images were taken. For Alizarin Red staining, formalin-fixed cells were incubated with 2% Alizarin Red for 1 h (#A5533, Sigma), rinsed with H_2_O and images were captured. Proteoglycan synthesis was evaluated using Alcian Blue staining. Cells were fixed in 4% PFA and stained with 0.1% Alcian Blue in 0.1 N HCl overnight. Cells were washed and stored in 70% EtOH for image capturing. Alcian Blue was extracted using 6 M guanine-HCl and the OD was measured at 630 nm. Lipid droplet formation was assessed by Oil Red O staining. Formalin-fixed cells were rinsed in 60% isopropanol for 2 minutes. Subsequently, cells were incubated in Oil Red O staining solution (Sigma) prepared following the instruction of the manufacturer for 30 minutes, then rinsed with H_2_O before images were taken. Oil Red O was extracted by addition of isopropanol containing 4% NP40 and the OD was measured at 540 nm.

### Retroviral infection

pQCXIP-CD2-FAK was generated by cloning CD2-FAK from pLV-neo-CD2-FAK (Addgene) into the pQXCIP vector. PlatE cells were transfected with 9 μg empty pQCXIP vector, or with vectors containing CD-2FAK, ASAP1c or ASAP1-R811A [32] using FuGENE (Promega). Supernatant containing retroviruses were transferred to *Asap1*^*GT*/GT^ MEFs of passage 1 or 2. After selection with 0.5 μg/ml puromycin, cells were plated for adipogenic differentiation as described above.

### RNA isolation and qPCR

RNA was isolated using Trizol (Invitrogen) according to the manufacturer’s protocol. Subsequently, 1.5–2 μg of RNA was digested with 5 U DNaseI (Thermo Fisher Scientific, Waltham, MA, USA) for 30 min at 37°C. The reaction was stopped by addition of EDTA and heat inactivation, then the RNA was transcribed into cDNA using reverse transcriptase (Thermo Fisher Scientific) according to manufacturer’s protocol. Gene expression was analyzed using SYBR-Green mix (Applied Biosciences, Waltham, MA, USA) to perform real-time qPCR using the One Step Plus Realtime PCR System (Applied Biosciences) under the following PCR conditions: 15 sec 95°C, 1 min 60°C, 1 min 72°C. The ribosomal protein PO (RibPO) was used as a control to normalize the data. The primer pairs used to amplify the indicated cDNAs are listed in S1 Table.

### Western blot analysis

Mouse tissues and cells for protein lysates were lysed in RIPA buffer, homogenized by a Tissue LyserII (Qiagen, Venlo, Netherlands) and quantified using the BCA Protein Assay Kit (Thermo Fisher Scientific). Lysates were subjected to 8% or 10% SDS-PAGE and transferred to a PVDF membrane. Protein expression was analyzed using rabbit anti-ASAP1 (ab12423, 1:2500, Abcam, Cambridge, UK), rabbit anti-FAK (06–543, 1:1000, Merck Milipore, Darmstadt, Germany), rabbit anti-phospho FAK Y397 (44-624G, 1:1000, Thermofisher), rabbit anti-Src (ab47405, 1:1000, abcam), rabbit anti-phospho Src Y416 (#6943, 1:1000, Cell Signaling), rabbit anti-phospho AKT T308 (#9275, 1:1000, Cell Signaling), rabbit anti-AKT (sc-8312, 1, 1:200, Santa Cruz), rabbit anti-ERK (sc-93, 1:200, Santa Cruz), mouse anti-phospho ERK (#9106, 1:1000, Cell Signaling), rabbit anti-p38 (sc535, 1:200, Santa Cruz), rabbit anti-phospho p38 (#4511, 1:1000, Cell Signaling). Probing the membranes with mouse anti-vinculin antibodies (V4139, 1:5000, Sigma) served as a loading control.

### Statistics

Differences between two groups were analyzed by a two-tailed unpaired Student’s t-test using Graphpad Prism. P values < 0.05 were considered statistically significant and indicated as follow: * p < 0.05, ** p < 0.005, *** p < 0.005.

## Supporting information

S1 Fig Characterization of ASAP1 gene-trap mice. (**A**) Insertion of the gene-trap vector into the ASAP1 gene locus was verified by genotyping PCR. Three primers were designed that specifically bind to either genomic ASAP1 sequences (*Asap1*_for; *Asap1*_rev) or to a sequence specific for the Gene-trap vector (*Gene-trap*_for). *Asap1*_rev primer binds to a portion of the ASAP1 genomic sequence flanking the gene-trap insertion site, and therefore binds to both the WT and GT genotypes. *Asap1*_for binds to a portion of the genomic sequence deleted in GT animals. The *Asap1*_for and *Asap1*_rev primer pair specifically amplifies a 330 bp amplicon when a wild-type allele is present. The *Gene-trap*_for primer together with the *Asap1*_rev primers amplifies a 680 bp amplicon only in the knock-out allele, when the gene-trap vector is present. A representative result of a PCR in which examples for genotyping of *Asap1*^+/+^, *Asap1*^+/GT^ and *Asap1*^*GT*/GT^ mice are shown is depicted (lower panel). Water instead of genomic DNA served as a negative control. (**B**) 3’-terminal parts of ASAP1 are not expressed upon gene-trap vector insertion. qPCR analysis using different primer pairs targeting exons 11–13, exons 21–24 or exons 29–30 of the ASAP1 gene locus was performed with cDNA from *Asap1*^+/+^ and *Asap1*^*GT*/GT^ MEFs. **(C)** Table showing Mendelian distribution of FVB; Asap1 progeny. *Asap1*^+/GT^ mice were intercrossed and progeny was genotyped at the age 10 days.
(TIF)

S2 Fig ASAP1 is expressed in adult organs. (**A-H**) X-Gal staining of trachea (**A**), heart (**B**), brain (**C**), bone (**D**), lung (**E, F**) and spleen (**G, H**). (**A-D, F, H**) Organs from an *Asap1*^*GT*/GT^ adult mouse. (**E, G**) Organs from an *Asap1*^*+/+*^ adult mouse. (**I**) Western blot analysis of different organs isolated from *Asap1*^+/+^ and *Asap1*^*GT*/GT^ adult mice. Protein lysates were prepared from heart, lung, liver, brain and testis and ASAP1 expression was detected using ASAP1 antibody. Vinculin served as loading control.
(TIF)

S3 Fig ASAP1 does not affect retinal angiogenesis and lymphangiogenesis. (**A**) Representative pictures of stained retinas from *Asap1*^+/+^ and *Asap1*^*GT*/GT^ neonates at P7 (**B**) Quantification of the angiogenic area of stained *Asap1*^+/+^ and *Asap1*^*GT*/GT^ retinas (n = 3, n = 4). (**C**) Analysis of ASAP1 mRNA expression in *Asap1*^+/+^ lung, *Asap1*^*GT*/GT^ tissue and *Asap1*^+/+^ thoracic duct using semi-quantitative PCR. RT-, RNA without added reverse trancriptase, and water served as control. (**D**) No significant difference was observed in lymphatic vessel density in the skin from *Asap1*^+/+^ and *Asap1*^*GT*/GT^ mice (n = 6, n = 6, p = 0.5). (**E**) No significant difference was observed in lymphangiogenesis in thoracic duct ring assays using thoracic duct from *Asap1*^+/+^ and *Asap1*^*GT*/GT^ mice (n = 30, n = 54, p = 0.7).
(TIF)

S4 Fig Focal adhesion formation and adhesion capacity is not impaired by loss of ASAP. WT (**A, C, E**) and *Asap1*^*GT/GT*^ MEFs (**B, D, F**) were plated on gelatin-coated cover slips and were either treated for 1h or for 24 h with adipogenic differentiation cocktail, or left untreated (control). Cells were stained using anti-vinculin (in green) and anti-phospho-FAK antibodies (in red). Scale bars: 50μm. (**G**) Adhesion of WT and *Asap1*^*GT/GT*^ MEFs to tissue culture plastic coated with fibronectin, laminin and collagen I. Cells were allowed to adhere for 1h at 37°C. Adhered cells were stained with crystal violet. Bound dye was extracted using 10% acetic acid and absorbance was measured at 595 nm. Data are presented as the mean +/- SE of triplicate samples.
(TIF)

S1 Table Primer sets used in qPCR. (PDF)

S1 File Supplementary methods. (PDF)

S1 Data Underlying numerical data. File of raw data underlying graphs in main figures.
(XLSX)

## References

[1] Ghaben AL, Scherer PE. Adipogenesis and metabolic health. Nat Rev Mol Cell Biol. 2019 10.1038/s41580-018-0093-z . 30610207

[2] Infante A, Rodriguez CI. Osteogenesis and aging: lessons from mesenchymal stem cells. Stem Cell Res Ther. 2018;9(1):244 10.1186/s13287-018-0995-x 30257716 PMC6158877

[3] Hammarstedt A, Gogg S, Hedjazifar S, Nerstedt A, Smith U. Impaired Adipogenesis and Dysfunctional Adipose Tissue in Human Hypertrophic Obesity. Physiol Rev. 2018;98(4):1911–41. 10.1152/physrev.00034.2017 . 30067159

[4] Nakashima K, Zhou X, Kunkel G, Zhang Z, Deng JM, Behringer RR, et al The novel zinc finger-containing transcription factor osterix is required for osteoblast differentiation and bone formation. Cell. 2002;108(1):17–29. . 11792318 10.1016/s0092-8674(01)00622-5

[5] Sinha KM, Zhou X. Genetic and molecular control of osterix in skeletal formation. J Cell Biochem. 2013;114(5):975–84. 10.1002/jcb.24439 23225263 PMC3725781

[6] Akiyama H, Chaboissier MC, Martin JF, Schedl A, de Crombrugghe B. The transcription factor Sox9 has essential roles in successive steps of the chondrocyte differentiation pathway and is required for expression of Sox5 and Sox6. Genes Dev. 2002;16(21):2813–28. 10.1101/gad.1017802 12414734 PMC187468

[7] Bi W, Deng JM, Zhang Z, Behringer RR, de Crombrugghe B. Sox9 is required for cartilage formation. Nat Genet. 1999;22(1):85–9. 10.1038/8792 . 10319868

[8] Long F, Ornitz DM. Development of the endochondral skeleton. Cold Spring Harb Perspect Biol. 2013;5(1):a008334 10.1101/cshperspect.a008334 23284041 PMC3579395

[9] Rosen ED, Spiegelman BM. What we talk about when we talk about fat. Cell. 2014;156(1–2):20–44. 10.1016/j.cell.2013.12.012 24439368 PMC3934003

[10] Tontonoz P, Hu E, Spiegelman BM. Stimulation of adipogenesis in fibroblasts by PPAR gamma 2, a lipid-activated transcription factor. Cell. 1994;79(7):1147–56. . 8001151 10.1016/0092-8674(94)90006-x

[11] Wang F, Mullican SE, DiSpirito JR, Peed LC, Lazar MA. Lipoatrophy and severe metabolic disturbance in mice with fat-specific deletion of PPARgamma. Proc Natl Acad Sci U S A. 2013;110(46):18656–61. 10.1073/pnas.1314863110 24167256 PMC3831974

[12] Hall BK, Miyake T. All for one and one for all: condensations and the initiation of skeletal development. Bioessays. 2000;22(2):138–47. 10.1002/(SICI)1521-1878(200002)22:2<138::AID-BIES5>3.0.CO;2-4 . 10655033

[13] Maes C. Signaling pathways effecting crosstalk between cartilage and adjacent tissues: Seminars in cell and developmental biology: The biology and pathology of cartilage. Semin Cell Dev Biol. 2017;62:16–33. 10.1016/j.semcdb.2016.05.007 . 27180955

[14] Lee JS, Ha L, Kwon IK, Lim JY. The role of focal adhesion kinase in BMP4 induction of mesenchymal stem cell adipogenesis. Biochem Biophys Res Commun. 2013;435(4):696–701. 10.1016/j.bbrc.2013.05.045 . 23702483

[15] Sun Y, Ma YC, Huang J, Chen KY, McGarrigle DK, Huang XY. Requirement of SRC-family tyrosine kinases in fat accumulation. Biochemistry. 2005;44(44):14455–62. 10.1021/bi0509090 . 16262245

[16] Rajshankar D, Wang Y, McCulloch CA. Osteogenesis requires FAK-dependent collagen synthesis by fibroblasts and osteoblasts. FASEB J. 2017;31(3):937–53. 10.1096/fj.201600645R . 27881487

[17] Marzia M, Sims NA, Voit S, Migliaccio S, Taranta A, Bernardini S, et al Decreased c-Src expression enhances osteoblast differentiation and bone formation. J Cell Biol. 2000;151(2):311–20. 10.1083/jcb.151.2.311 11038178 PMC2192638

[18] Pala D, Kapoor M, Woods A, Kennedy L, Liu S, Chen S, et al Focal adhesion kinase/Src suppresses early chondrogenesis: central role of CCN2. J Biol Chem. 2008;283(14):9239–47. 10.1074/jbc.M705175200 18276598 PMC2431031

[19] Shin H, Lee MN, Choung JS, Kim S, Choi BH, Noh M, et al Focal Adhesion Assembly Induces Phenotypic Changes and Dedifferentiation in Chondrocytes. J Cell Physiol. 2016;231(8):1822–31. 10.1002/jcp.25290 . 26661891

[20] Schaller MD, Hildebrand JD, Shannon JD, Fox JW, Vines RR, Parsons JT. Autophosphorylation of the focal adhesion kinase, pp125FAK, directs SH2-dependent binding of pp60src. Mol Cell Biol. 1994;14(3):1680–8. 10.1128/mcb.14.3.1680 7509446 PMC358526

[21] Parsons JT. Focal adhesion kinase: the first ten years. J Cell Sci. 2003;116(Pt 8):1409–16. 10.1242/jcs.00373 . 12640026

[22] Chen HC, Guan JL. Association of focal adhesion kinase with its potential substrate phosphatidylinositol 3-kinase. Proc Natl Acad Sci U S A. 1994;91(21):10148–52. 10.1073/pnas.91.21.10148 7937853 PMC44975

[23] Brown MT, Andrade J, Radhakrishna H, Donaldson JG, Cooper JA, Randazzo PA. ASAP1, a phospholipid-dependent arf GTPase-activating protein that associates with and is phosphorylated by Src. Mol Cell Biol. 1998;18(12):7038–51. 10.1128/mcb.18.12.7038 9819391 PMC109286

[24] Furman C, Short SM, Subramanian RR, Zetter BR, Roberts TM. DEF-1/ASAP1 is a GTPase-activating protein (GAP) for ARF1 that enhances cell motility through a GAP-dependent mechanism. J Biol Chem. 2002;277(10):7962–9. 10.1074/jbc.M109149200 . 11773070

[25] Onodera Y, Hashimoto S, Hashimoto A, Morishige M, Mazaki Y, Yamada A, et al Expression of AMAP1, an ArfGAP, provides novel targets to inhibit breast cancer invasive activities. EMBO J. 2005;24(5):963–73. 10.1038/sj.emboj.7600588 15719014 PMC554134

[26] Liu Y, Loijens JC, Martin KH, Karginov AV, Parsons JT. The association of ASAP1, an ADP ribosylation factor-GTPase activating protein, with focal adhesion kinase contributes to the process of focal adhesion assembly. Mol Biol Cell. 2002;13(6):2147–56. 10.1091/mbc.E02-01-0018 12058076 PMC117631

[27] Tien DN, Kishihata M, Yoshikawa A, Hashimoto A, Sabe H, Nishi E, et al AMAP1 as a negative-feedback regulator of nuclear factor-kappaB under inflammatory conditions. Sci Rep. 2014;4:5094 10.1038/srep05094 24865276 PMC4035583

[28] Randazzo PA, Andrade J, Miura K, Brown MT, Long YQ, Stauffer S, et al The Arf GTPase-activating protein ASAP1 regulates the actin cytoskeleton. Proc Natl Acad Sci U S A. 2000;97(8):4011–6. 10.1073/pnas.070552297 10725410 PMC18133

[29] Bharti S, Inoue H, Bharti K, Hirsch DS, Nie Z, Yoon HY, et al Src-dependent phosphorylation of ASAP1 regulates podosomes. Mol Cell Biol. 2007;27(23):8271–83. 10.1128/MCB.01781-06 17893324 PMC2169185

[30] Liu Y, Yerushalmi GM, Grigera PR, Parsons JT. Mislocalization or reduced expression of Arf GTPase-activating protein ASAP1 inhibits cell spreading and migration by influencing Arf1 GTPase cycling. J Biol Chem. 2005;280(10):8884–92. 10.1074/jbc.M412200200 . 15632162

[31] Onodera Y, Nam JM, Hashimoto A, Norman JC, Shirato H, Hashimoto S, et al Rab5c promotes AMAP1-PRKD2 complex formation to enhance beta1 integrin recycling in EGF-induced cancer invasion. J Cell Biol. 2012;197(7):983–96. 10.1083/jcb.201201065 22734003 PMC3384417

[32] Muller T, Stein U, Poletti A, Garzia L, Rothley M, Plaumann D, et al ASAP1 promotes tumor cell motility and invasiveness, stimulates metastasis formation in vivo, and correlates with poor survival in colorectal cancer patients. Oncogene. 2010;29(16):2393–403. 10.1038/onc.2010.6 . 20154719

[33] Ehlers JP, Worley L, Onken MD, Harbour JW. DDEF1 is located in an amplified region of chromosome 8q and is overexpressed in uveal melanoma. Clin Cancer Res. 2005;11(10):3609–13. 10.1158/1078-0432.CCR-04-1941 . 15897555

[34] Lin D, Watahiki A, Bayani J, Zhang F, Liu L, Ling V, et al ASAP1, a gene at 8q24, is associated with prostate cancer metastasis. Cancer Res. 2008;68(11):4352–9. 10.1158/0008-5472.CAN-07-5237 . 18519696

[35] Gossler A, Joyner AL, Rossant J, Skarnes WC. Mouse embryonic stem cells and reporter constructs to detect developmentally regulated genes. Science. 1989;244(4903):463–5. 10.1126/science.2497519 . 2497519

[36] Stryke D, Kawamoto M, Huang CC, Johns SJ, King LA, Harper CA, et al BayGenomics: a resource of insertional mutations in mouse embryonic stem cells. Nucleic Acids Res. 2003;31(1):278–81. 10.1093/nar/gkg064 12520002 PMC165511

[37] Hashimoto A, Hashimoto S, Ando R, Noda K, Ogawa E, Kotani H, et al GEP100-Arf6-AMAP1-cortactin pathway frequently used in cancer invasion is activated by VEGFR2 to promote angiogenesis. PLoS One. 2011;6(8):e23359 10.1371/journal.pone.0023359 21858086 PMC3156124

[38] King FJ, Hu E, Harris DF, Sarraf P, Spiegelman BM, Roberts TM. DEF-1, a novel Src SH3 binding protein that promotes adipogenesis in fibroblastic cell lines. Mol Cell Biol. 1999;19(3):2330–7. 10.1128/mcb.19.3.2330 10022919 PMC84025

[39] Peng XD, Xu PZ, Chen ML, Hahn-Windgassen A, Skeen J, Jacobs J, et al Dwarfism, impaired skin development, skeletal muscle atrophy, delayed bone development, and impeded adipogenesis in mice lacking Akt1 and Akt2. Genes Dev. 2003;17(11):1352–65. 10.1101/gad.1089403 12782654 PMC196068

[40] Prusty D, Park BH, Davis KE, Farmer SR. Activation of MEK/ERK signaling promotes adipogenesis by enhancing peroxisome proliferator-activated receptor gamma (PPARgamma) and C/EBPalpha gene expression during the differentiation of 3T3-L1 preadipocytes. J Biol Chem. 2002;277(48):46226–32. 10.1074/jbc.M207776200 . 12270934

[41] Xu J, Liao K. Protein kinase B/AKT 1 plays a pivotal role in insulin-like growth factor-1 receptor signaling induced 3T3-L1 adipocyte differentiation. J Biol Chem. 2004;279(34):35914–22. 10.1074/jbc.M402297200 . 15192111

[42] Mukherjee A, Rotwein P. Akt promotes BMP2-mediated osteoblast differentiation and bone development. J Cell Sci. 2009;122(Pt 5):716–26. 10.1242/jcs.042770 19208758 PMC2720922

[43] Mauvais-Jarvis F, Arnold AP, Reue K. A Guide for the Design of Pre-clinical Studies on Sex Differences in Metabolism. Cell Metab. 2017;25(6):1216–30. 10.1016/j.cmet.2017.04.033 28591630 PMC5516948

[44] Stubbins RE, Holcomb VB, Hong J, Nunez NP. Estrogen modulates abdominal adiposity and protects female mice from obesity and impaired glucose tolerance. Eur J Nutr. 2012;51(7):861–70. 10.1007/s00394-011-0266-4 . 22042005

[45] Peruzzi B, Cappariello A, Del Fattore A, Rucci N, De Benedetti F, Teti A. c-Src and IL-6 inhibit osteoblast differentiation and integrate IGFBP5 signalling. Nat Commun. 2012;3:630 10.1038/ncomms1651 . 22252554

[46] Kita K, Kimura T, Nakamura N, Yoshikawa H, Nakano T. PI3K/Akt signaling as a key regulatory pathway for chondrocyte terminal differentiation. Genes Cells. 2008;13(8):839–50. 10.1111/j.1365-2443.2008.01209.x . 18782222

[47] Campa F, Randazzo PA. Arf GTPase-activating proteins and their potential role in cell migration and invasion. Cell Adh Migr. 2008;2(4):258–62. 10.4161/cam.2.4.6959 19262159 PMC2601647

[48] Enomoto A, Murakami H, Asai N, Morone N, Watanabe T, Kawai K, et al Akt/PKB regulates actin organization and cell motility via Girdin/APE. Dev Cell. 2005;9(3):389–402. 10.1016/j.devcel.2005.08.001 . 16139227

[49] Mathieu PS, Loboa EG. Cytoskeletal and focal adhesion influences on mesenchymal stem cell shape, mechanical properties, and differentiation down osteogenic, adipogenic, and chondrogenic pathways. Tissue Eng Part B Rev. 2012;18(6):436–44. 10.1089/ten.TEB.2012.0014 22741572 PMC3495119

[50] Rodriguez JP, Gonzalez M, Rios S, Cambiazo V. Cytoskeletal organization of human mesenchymal stem cells (MSC) changes during their osteogenic differentiation. J Cell Biochem. 2004;93(4):721–31. 10.1002/jcb.20234 . 15660416

[51] Treiser MD, Yang EH, Gordonov S, Cohen DM, Androulakis IP, Kohn J, et al Cytoskeleton-based forecasting of stem cell lineage fates. Proc Natl Acad Sci U S A. 2010;107(2):610–5. 10.1073/pnas.0909597107 20080726 PMC2818905

[52] Boulay PL, Cotton M, Melancon P, Claing A. ADP-ribosylation factor 1 controls the activation of the phosphatidylinositol 3-kinase pathway to regulate epidermal growth factor-dependent growth and migration of breast cancer cells. J Biol Chem. 2008;283(52):36425–34. 10.1074/jbc.M803603200 18990689 PMC2662303

[53] Song M, Bode AM, Dong Z, Lee MH. AKT as a Therapeutic Target for Cancer. Cancer Res. 2019 10.1158/0008-5472.CAN-18-2738 . 30808672

[54] Li H, Durbin R. Fast and accurate short read alignment with Burrows-Wheeler transform. Bioinformatics. 2009;25(14):1754–60. 10.1093/bioinformatics/btp324 19451168 PMC2705234

[55] Thorvaldsdottir H, Robinson JT, Mesirov JP. Integrative Genomics Viewer (IGV): high-performance genomics data visualization and exploration. Brief Bioinform. 2013;14(2):178–92. 10.1093/bib/bbs017 22517427 PMC3603213

[56] Schatz O, Golenser E, Ben-Arie N. Clearing and photography of whole mount X-gal stained mouse embryos. Biotechniques. 2005;39(5):650, 2, 4 passim. 10.2144/000112034 . 16312214

