# Supplementary figures and images for: Loss of ASAP1 in mice impairs adipogenic and osteogenic differentiation of mesenchymal progenitor cells through dysregulation of FAK/Src and AKT signaling
Source: PLoS Genet. 2019 Jun 27;15(6):e1008216. doi: 10.1371/journal.pgen.1008216 (PMC6619832; doi:10.1371/journal.pgen.1008216)

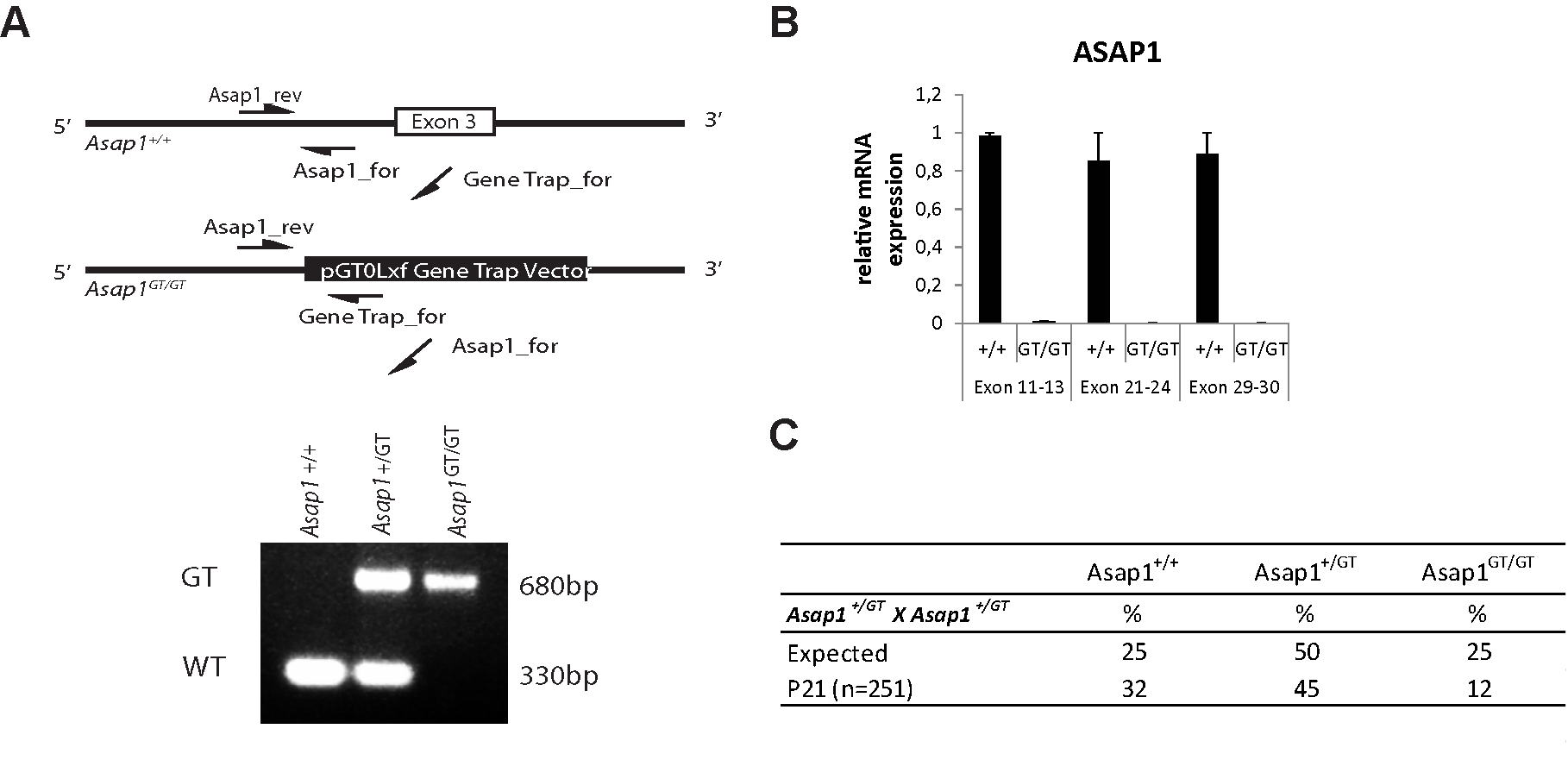

Supplement: S1 Fig — (A) Insertion of the gene-trap vector into the ASAP1 gene locus was verified by genotyping PCR. Three primers were designed that specifically bind to either genomic ASAP1 sequences (Asap1_for; Asap1_rev) or to a sequence specific for the Gene-trap vector (Gene-trap_for). Asap1_rev primer binds to a portion of the ASAP1 genomic sequence flanking the gene-trap insertion site, and therefore binds to both the WT and GT genotypes. Asap1_for binds to a portion of the genomic sequence deleted in GT animals. The Asap1_for and Asap1_rev primer pair specifically amplifies a 330 bp amplicon when a wild-type allele is present. The Gene-trap_for primer together with the Asap1_rev primers amplifies a 680 bp amplicon only in the knock-out allele, when the gene-trap vector is present. A representative result of a PCR in which examples for genotyping of Asap1+/+, Asap1+/GT and Asap1GT/GT mice are shown is depicted (lower panel). Water instead of genomic DNA served as a negative control. (B) 3’-terminal parts of ASAP1 are not expressed upon gene-trap vector insertion. qPCR analysis using different primer pairs targeting exons 11–13, exons 21–24 or exons 29–30 of the ASAP1 gene locus was performed with cDNA from Asap1+/+ and Asap1GT/GT MEFs. (C) Table showing Mendelian distribution of FVB; Asap1 progeny. Asap1+/GT mice were intercrossed and progeny was genotyped at the age 10 days. (TIF) [file pgen.1008216.s001.tif]

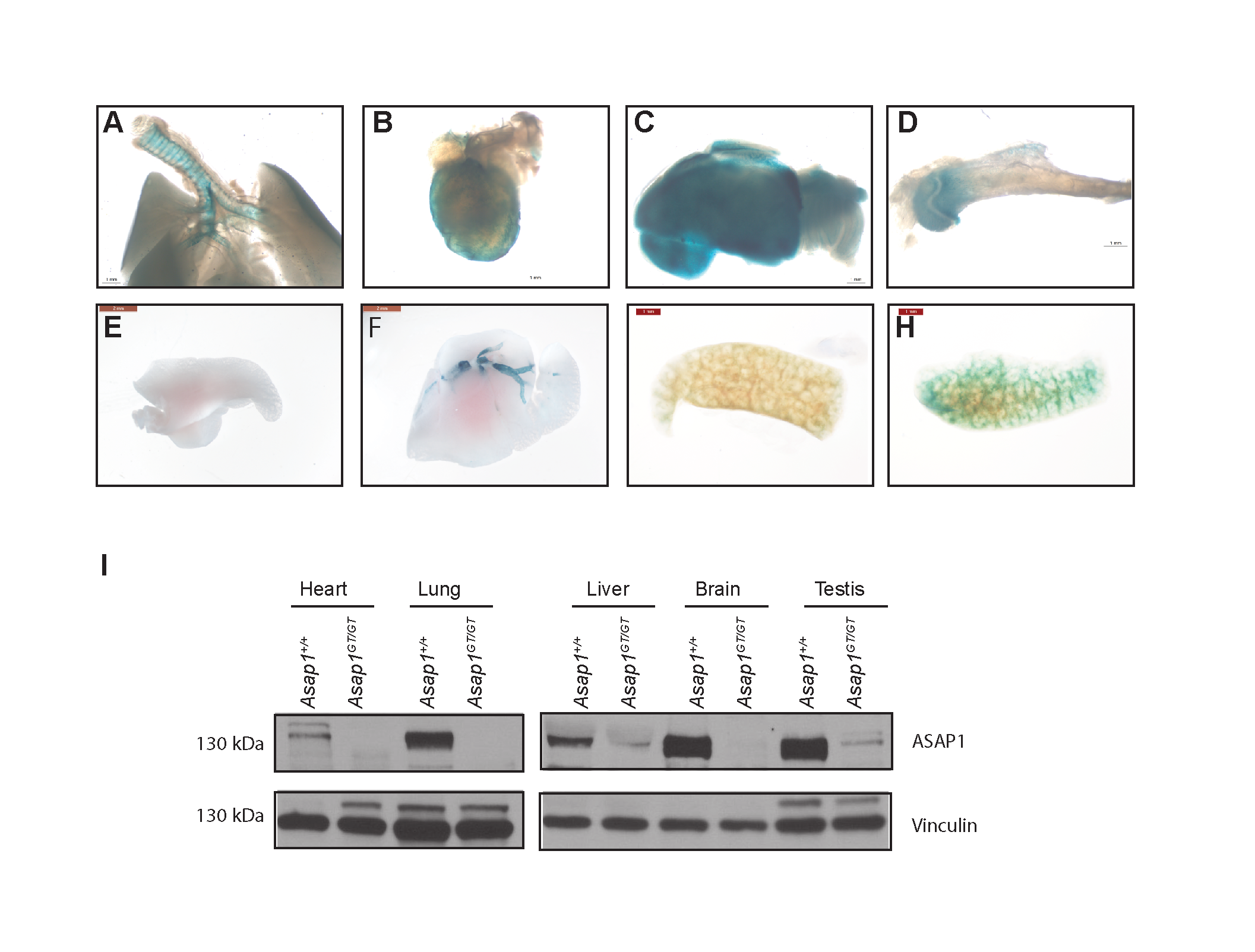

Supplement: S2 Fig — (A-H) X-Gal staining of trachea (A), heart (B), brain (C), bone (D), lung (E, F) and spleen (G, H). (A-D, F, H) Organs from an Asap1GT/GT adult mouse. (E, G) Organs from an Asap1+/+ adult mouse. (I) Western blot analysis of different organs isolated from Asap1+/+ and Asap1GT/GT adult mice. Protein lysates were prepared from heart, lung, liver, brain and testis and ASAP1 expression was detected using ASAP1 antibody. Vinculin served as loading control. (TIF) [file pgen.1008216.s002.tif]

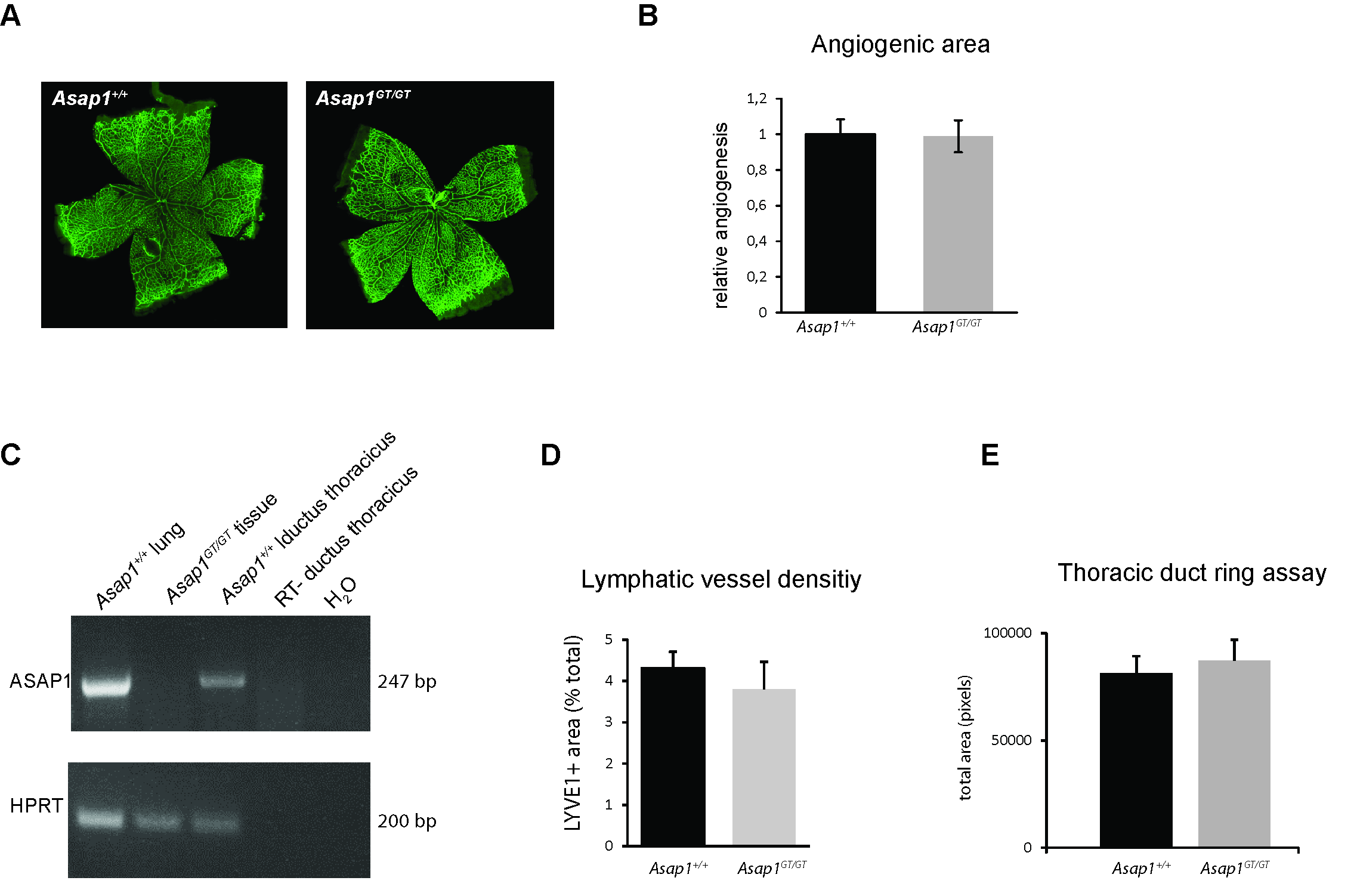

Supplement: S3 Fig — (A) Representative pictures of stained retinas from Asap1+/+ and Asap1GT/GT neonates at P7 (B) Quantification of the angiogenic area of stained Asap1+/+ and Asap1GT/GT retinas (n = 3, n = 4). (C) Analysis of ASAP1 mRNA expression in Asap1+/+ lung, Asap1GT/GT tissue and Asap1+/+ thoracic duct using semi-quantitative PCR. RT-, RNA without added reverse trancriptase, and water served as control. (D) No significant difference was observed in lymphatic vessel density in the skin from Asap1+/+ and Asap1GT/GT mice (n = 6, n = 6, p = 0.5). (E) No significant difference was observed in lymphangiogenesis in thoracic duct ring assays using thoracic duct from Asap1+/+ and Asap1GT/GT mice (n = 30, n = 54, p = 0.7). (TIF) [file pgen.1008216.s003.tif]

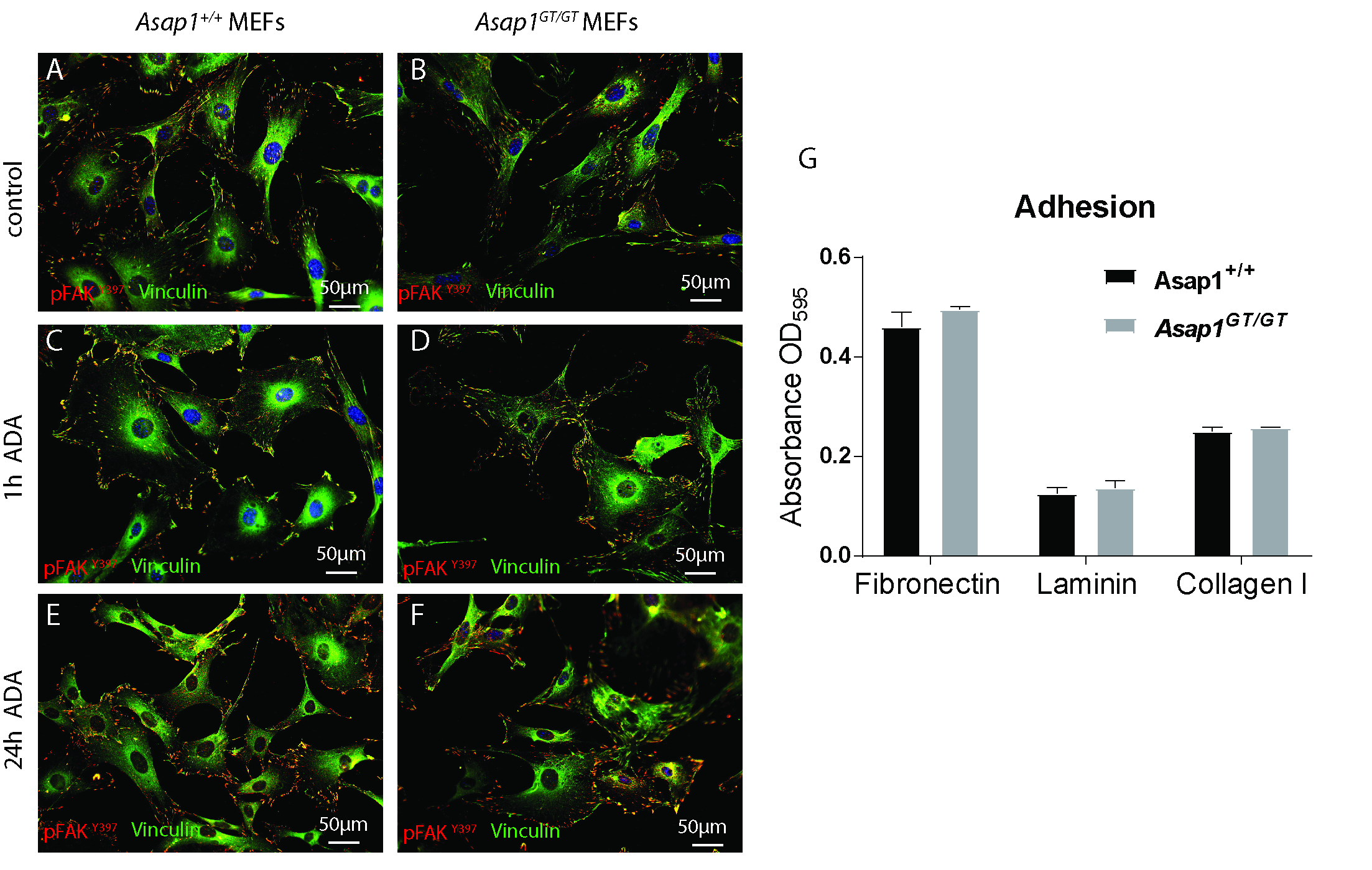

Supplement: S4 Fig — WT (A, C, E) and Asap1GT/GT MEFs (B, D, F) were plated on gelatin-coated cover slips and were either treated for 1h or for 24 h with adipogenic differentiation cocktail, or left untreated (control). Cells were stained using anti-vinculin (in green) and anti-phospho-FAK antibodies (in red). Scale bars: 50μm. (G) Adhesion of WT and Asap1GT/GT MEFs to tissue culture plastic coated with fibronectin, laminin and collagen I. Cells were allowed to adhere for 1h at 37°C. Adhered cells were stained with crystal violet. Bound dye was extracted using 10% acetic acid and absorbance was measured at 595 nm. Data are presented as the mean +/- SE of triplicate samples. (TIF) [file pgen.1008216.s004.tif]
