# Supplementary material for: Loss of ASAP1 in mice impairs adipogenic and osteogenic differentiation of mesenchymal progenitor cells through dysregulation of FAK/Src and AKT signaling
Source: PLoS Genet. 2019 Jun 27;15(6):e1008216. doi: 10.1371/journal.pgen.1008216 (PMC6619832; doi:10.1371/journal.pgen.1008216)
Supplement: S1 Table — (PDF) [file pgen.1008216.s005.pdf]

**Name of Genes**	**Forward primer (5'-3')**	**Reverse primer (5'-3')**
**m*RibPO***	GGACCCGAGAAGACCTCCTT	gcacatcactcagaatttcaatgg
**m*Asap1* Exon 11-13**	TGGTTTATATGTTGCCAGCAGGGC	TTTCACTGCCGTACTCCTTGTTGC
**m*Asap1* Exon 21-24**	ACTATTGCAGCATGTACGGCAAGC	TGGGAAAGCAGATCTTCACACTGG
**m*Asap1* Exon 29-30**	AGCAAGCATCTGAAGACTCCAACG	AGTCATAAATGGTCTTCACCCGCC
**m*Acan***	AGG ACC TGG TAG TGC GAG TG	GCG TGT GGC GAA GAA
**m*Ihh***	TCA CCC CCA ACT ACA ATC CC	CCG TGT TCT CCT CGT CCT TG
**m*Runx2***	TAA AGT GAC AGT GGA CGG TCC C	TGC GCC CTA AAT CAC TGA GG
**m*Osx***	AGCGACCACTTGAGCAAACAT	GCGGCTGATTGGCTTCTTCT
**m*BSP***	CCAGGACTGCCGAAAGGAAG	CCCCGTTTTCTTCAGAATCCTCTG
**m*OCN***	CTG ACA AAG CCT TCA TGT CCA A	GCG CCG GAG TCT GTT CAC TA
**m*Cebpb***	CAAGCTGAGCGACGAGTACA	CAGCTGCTCCACCTTCTTCT
**m*Cebpa***	GTGCTGGAGTTGACCAGTGA	AAACCATCCTCTGGGTCTCC
**m*Pparg***	GGGGTGATGTGTTTGAACTTG	CAGGAAAGACAACAGACAAAT
**m*Slc2a4***	TGTCGCTGGTTTCTCCAACTG	CCATAGCATCCGCAACATACTG
**m*Adipoq***	CCTGGCCACTTTCTCCTCATT	ACAGGAGAGCTTGCAACAGT
