# Supplementary material for: Loss of ASAP1 in mice impairs adipogenic and osteogenic differentiation of mesenchymal progenitor cells through dysregulation of FAK/Src and AKT signaling
Source: PLoS Genet. 2019 Jun 27;15(6):e1008216. doi: 10.1371/journal.pgen.1008216 (PMC6619832; doi:10.1371/journal.pgen.1008216)
Supplement: S1 File — (PDF) [file pgen.1008216.s006.pdf]

S1 Supplementary methods

Retina whole-mount staining

Retina whole-mount staining was performed as described (1). Briefly, at P7, wild-type and Asap1$^{GT/GT}$ mice were sacrificed, eyeballs were isolated and fixed in ice-cold methanol. Retinas were isolated, and blocking was performed with 0.5% Triton X-100/ 1% bovine serum albumin/ 10% goat serum in PBS for 30 min at room temperature. The retinal vasculature was stained with FITC-conjugated Isolectin B-4 (IB-4, Sigma L9381; 1:100) overnight at 4°C. Pictures were taken with the confocal microscope Zeiss LSM710 and image analysis was accomplished with ImageJ. The relative vessel area was calculated as IB-4^{+} area per retina area. Values are normalized relative to the mean of the wild-type animals.

Thoracic duct ring cultures

Lymphatic ring cultures were performed as described previously (2). Briefly, thoracic ducts were dissected from wild-type or Asap1^{GT}/^{GT} mice and cut into 1 mm long pieces. The explants were embedded in rat tail interstitial collagen gel (Serva Electrophoresis) and then polymerized in cylindrical agarose wells. They were then cultivated for 13 days. To quantify vessel outgrowth, binary images of photographs taken under identical conditions of light, contrast and magnification were analyzed using NIH ImageJ software.

Whole mount lymphatic vessel staining of mouse skin

The skin was shaved and excised. Fat and connective tissue were gently removed with scissors and skin samples were put in 0.25%-Trypsin/ EDTA overnight at 4°C. The next day digested epidermis was removed using forceps and the skin was horizontally fixed on a

paraffin bed with fine insect needles. After several short washes with PBS and fixation with 4% PFA overnight at 4°C, skin samples were placed into 10% goat serum/ PBS/ 0.3% Triton X-100 over night at 4°C to permeabilize the skin and block unspecific binding sites. Subsequently rabbit anti-mouse Lyve1 antibodies (cat# 103-PA50, Reliatech, Germany) were diluted at 3 µg/ml in 10% goat serum/ PBS/ 0.3% Triton X-100 and incubated with the samples overnight at 4°C. Samples were then washed six times for 30 minutes at room temperature with PBS/ 0.3% Triton X-100 before incubation at 4°C overnight with secondary goat anti-rabbit Alexa 546 antibodies (Invitrogen, Germany). Unbound antibodies were washed off.

In order to clear the skin, samples were dehydrated with graded steps of ethanol. After dehydration samples were cleared in methylsalicilate (Oil-of-wintergreen, Sigma, Germany) in glass vials and kept at room temperature protected from light. Pictures were taken at 1.6x magnification using a fluorescence stereomicroscope (Leica, Germany). Quantification of lymphatic vasculature density was performed for five independent 1 mm^2 fields of each sample using Image J.

Immunoflorescence staining of MEFs

WT and Asap1$^{GT/GT}$ MEFs were plated on gelatin-coated cover slips. After 24h, cells were treated for 1h or 24h with adipogenic differentiation cocktail or were left untreated. After fixation with 4% PFA, cells were permeabilized with 0.1% Triton X for 10 min, washed with PBS and blocked with 10% goat serum, 5% FCS, and 0.5% BSA for 1h. Sections were incubated with anti-vinculin (V4139, 1: 200, Sigma) and anti-phospho FAK Y397 (44624G, 1:200, Thermofisher) antibodies in blocking solution for 1h. After washing, sections were incubated

for 45 min in the dark with secondary anti-rabbit Alexa 546 and antimouse Alexa 488 antibodies, then counterstained with DAPI. Staining was analyzed using a Zeiss Axio Imager.

Adhesion assays

Coating of 96-well plates with 20 µg/ ml fibronectin, 20 µg /ml collagen I, or 20 µg/ ml laminin was performed at 37°C for 1h. Wells were washed twice with PBS, blocked with 1% BSA for 30 min 37°C, then washed again in PBS. WT and Asap1$^{GT/GT}$ MEFs were trypsinized and $5x10^4$ cells were seeded per well in serum-free DMEM in triplicates. After incubation for 1h at 37°C, adhered cells were fixed with 4% PFA for 10 min, then stained with crystal violet for 30 min. Bound dye was extracted using 10% acetic acid and absorbance was measured at 595 nm.

References

1. Korn C, Scholz B, Hu J, Srivastava K, Wojtarowicz J, Arnsperger T, et al. Endothelial cell-derived non-canonical Wnt ligands control vascular pruning in angiogenesis. Development. 2014;141(8):1757-66.

2. Thiele W, Rothley M, Teller N, Jung N, Bulat B, Plaumann D, et al. Delphinidin is a novel inhibitor of lymphangiogenesis but promotes mammary tumor growth and metastasis formation in syngeneic experimental rats. Carcinogenesis. 2013;34(12):2804-13.
